# The Health System and Population Health Implications of Large-Scale Diabetes Screening in India: A Microsimulation Model of Alternative Approaches

**DOI:** 10.1371/journal.pmed.1001827

**Published:** 2015-05-19

**Authors:** Sanjay Basu, Christopher Millett, Sandeep Vijan, Rodney A. Hayward, Sanjay Kinra, Rahoul Ahuja, John S. Yudkin

**Affiliations:** 1 Prevention Research Center, Centers for Health Policy, Primary Care and Outcomes Research, Center on Poverty and Inequality, and Cardiovascular Institute, Stanford University, Stanford, California, United States of America; 2 Department of Public Health and Policy, London School of Hygiene and Tropical Medicine, London, United Kingdom; 3 School of Public Health, Imperial College London, London, United Kingdom; 4 Public Health Foundation of India, Delhi, India; 5 Center for Clinical Management Research, Ann Arbor Veterans Affairs Hospital, Ann Arbor, Michigan, United States of America; 6 Department of Internal Medicine, University of Michigan, Ann Arbor, Michigan, United States of America; 7 Department of Epidemiology and Population Health, London School of Hygiene and Tropical Medicine, London, United Kingdom; 8 Division of Medicine, University College London, London, United Kingdom; Imperial College London, UNITED KINGDOM

## Abstract

**Background:**

Like a growing number of rapidly developing countries, India has begun to develop a system for large-scale community-based screening for diabetes. We sought to identify the implications of using alternative screening instruments to detect people with undiagnosed type 2 diabetes among diverse populations across India.

**Methods and Findings:**

We developed and validated a microsimulation model that incorporated data from 58 studies from across the country into a nationally representative sample of Indians aged 25–65 y old. We estimated the diagnostic and health system implications of three major survey-based screening instruments and random glucometer-based screening. Of the 567 million Indians eligible for screening, depending on which of four screening approaches is utilized, between 158 and 306 million would be expected to screen as “high risk” for type 2 diabetes, and be referred for confirmatory testing. Between 26 million and 37 million of these people would be expected to meet international diagnostic criteria for diabetes, but between 126 million and 273 million would be “false positives.” The ratio of false positives to true positives varied from 3.9 (when using random glucose screening) to 8.2 (when using a survey-based screening instrument) in our model. The cost per case found would be expected to be from US$5.28 (when using random glucose screening) to US$17.06 (when using a survey-based screening instrument), presenting a total cost of between US$169 and US$567 million. The major limitation of our analysis is its dependence on published cohort studies that are unlikely fully to capture the poorest and most rural areas of the country. Because these areas are thought to have the lowest diabetes prevalence, this may result in overestimation of the efficacy and health benefits of screening.

**Conclusions:**

Large-scale community-based screening is anticipated to produce a large number of false-positive results, particularly if using currently available survey-based screening instruments. Resource allocators should consider the health system burden of screening and confirmatory testing when instituting large-scale community-based screening for diabetes.

## Abstract 2

Sanjay Basu and colleagues estimate the benefits and costs of scaling up survey- or glucometer-based diabetes screening across India’s diverse populations.

## Editors' Summary

**Background:**

Worldwide, 387 million people have diabetes, a chronic condition characterized by high levels of glucose (sugar) in the blood. Blood sugar levels are usually controlled by insulin, a hormone released by the pancreas after meals. In people with type 2 diabetes (the most common type of diabetes), blood sugar control fails because the fat and muscle cells that normally respond to insulin by removing excess sugar from the blood become less responsive to insulin. Risk factors for diabetes include being overweight, having a large waist, being physically inactive, and having a family history of diabetes. The symptoms of diabetes, which develop slowly, include excessive urination at night and unexplained weight loss. Type 2 diabetes can usually be controlled initially with diet and exercise and with antidiabetic drugs such as metformin and sulfonylureas, but many patients eventually need insulin injections. Long-term complications of diabetes, which include an increased risk of heart disease and stroke, reduce the life expectancy of people with diabetes by about 10 years compared to people without diabetes.

**Why Was This Study Done?:**

Diabetes is becoming increasing common, particularly in rapidly developing countries, but most people with diabetes in these countries are unaware that they have the condition. Because the risk of developing diabetic complications is reduced by careful blood sugar control, it is important to identify and treat anyone who has diabetes as early as possible. Some rapidly developing countries are therefore beginning to develop systems for large-scale community-based screening for diabetes (even though the UK has recently decided against such screening). In India, for example, more than 53 million adults living in rural and urban communities have already been screened using either questionnaires designed to provide a risk score (survey-based screening) or random blood glucose testing (glucometer-based screening). People who are identified as “high risk” using these approaches are referred for fasting blood glucose tests to confirm the diagnosis. Although the Indian government plans to expand this screening program, no data have been collected to track its performance. Here, the researchers develop a microsimulation model (a computer model that operates at the level of individuals) to investigate the implications of using alternative screening instruments to identify people with undetected diabetes across diverse populations in India.

**What Did the Researchers Do and Find?:**

The researchers constructed a synthetic nationally representative population of Indians aged 25–65 years using data from 58 sub-national studies. They then used their microsimulation model to estimate the diagnostic and health system implications of using three survey-based screening instruments and glucometer-based screening to identify individuals in this population with diabetes. Depending on which approach was used for screening, between 158 million and 306 million of the 567 million Indians eligible for screening would be classified as high risk for diabetes and would be referred for confirmatory testing, according to the model. However, between 126 million and 273 million of these high-risk individuals would be false positives; only between 26 million and 37 million of these individuals would meet the international diagnostic criteria for diabetes (true positives). The researchers estimate that the cost per case found would vary from US$5.28 (when using random glucose screening) to US$17.06 (when using a survey-based screening instrument). Finally, they estimate that the total cost for screening the eligible population would be between US$169 and US$567 million.

**What Do These Findings Mean?:**

Established criteria for implementing screening programs specify that such programs should use reliable instruments that detect a large proportion of true cases (high sensitivity) and that have a low rate of false positives (high specificity). Screening programs should also offer significant therapeutic benefits to individuals diagnosed through screening. The findings of this study suggest that large-scale community-based screening for diabetes in India using the currently available screening instruments is unlikely to meet these criteria. Indeed, because the data used to construct the synthetic population came from published studies that did not capture the situation in the poorest, most rural areas of India, where the proportion of the population with diabetes is thought to be lowest, these findings may overestimate the efficacy and health benefits of screening. The researchers suggest, therefore, that an approach that focuses on symptom-based screening and on improvements in the treatment of already diagnosed individuals might be a more sensible path for India to take to deal with its burgeoning diabetes epidemic than community-based mass screening.

**Additional Information.:**

Please access these websites via the online version of this summary at http://dx.doi.org/10.1371/journal.pmed.1001827.

- The US National Diabetes Information Clearinghouse provides information about diabetes for patients, healthcare professionals, and the general public (in English and Spanish)
- The UK National Health Service Choices website provides information for patients and caregivers about type 2 diabetes and about living with diabetes; it also provides people’s stories about diabetes
- The charity Diabetes UK provides detailed information for patients and caregivers in several languages
- The UK-based non-profit organization HealthTalkOnline has interviews with people about their experiences of diabetes
- MedlinePlus provides links to further resources and advice about diabetes (in English and Spanish)
- A statement from the UK National Screening Committee on diabetes screening in adults is available

## Introduction

Type 2 diabetes has increased in prevalence at an alarming rate in rapidly developing countries such as India and China [1–4]. Most people with diabetes in these countries are undiagnosed; hence, community-based screening of adults for diabetes has been suggested [5–8]. In India, for example, a recently initiated program has already screened as many as 53 million adults in both urban and rural communities, using either survey-based instruments (i.e., risk-scoring questionnaires) or random (i.e., not necessarily fasting) blood glucose testing [9]. Individuals identified as high risk through these screening strategies are typically referred for fasting blood glucose tests to confirm the diagnosis. The Indian government plans to continue expanding this large-scale screening program in coming years. However, despite its potentially large impact, essentially no data have been collected to track the performance of the screening program [10].

Large-scale screening for diabetes, like population-wide screening for any disease, must fulfill several key criteria: (i) that a reliably sensitive and specific screening instrument is available, (ii) that facilities for diagnosis and treatment are available to those screened in order to initiate early therapy, (iii) that there is an agreed-upon policy on whom to treat among those screened, (iv) that the total cost of finding a case is included in estimating the impact of screening on medical expenditure as a whole, and (v) that beneficial early therapy delivered to those individuals newly diagnosed provides significant health advantages over the status quo [11]. In India, numerous survey-based screening instruments have been constructed to identify persons with a high risk of having undiagnosed diabetes among select sub-national Indian populations [6–8], yet these have not been tested more widely among diverse populations given the absence of large, nationally representative cohorts. The various risk factors incorporated into different instruments vary in prevalence among demographic populations and have very different associations with diabetes prevalence among urban and rural populations (e.g., [12]). Hence, screening instruments developed among some subpopulations may not be optimal for a standardized, national program. Furthermore, it remains unclear how many resources must be devoted to confirmatory testing and subsequent treatment to deliver population health benefits. In high-income countries, additional screening for high-risk, asymptomatic patients has not resulted in a significant reduction in all-cause, cardiovascular, or diabetes-related mortality, or in rates of diabetes-related microvascular complications such as blindness or renal failure [13,14].

We therefore constructed a microsimulation model to determine the implications of using alternative proposed screening instruments to identify persons with a high risk of having undiagnosed type 2 diabetes across diverse populations in India. We compared the three major survey-based screening instruments proposed for use in India [6–8], as well as a random glucometer-based screening approach that has been initiated by some government offices [15], to identify how different populations of people with diabetes in India would be detected through these alternative screening strategies. Both the questionnaire-based screening instruments and random glucometer-based screening are typically implemented through health “camps” that adopt a “come one, come all” strategy, whereby any members of the public can potentially be screened. We estimated rates of true- and false-positive and true- and false-negative screens, the associated need for confirmatory testing, and the implications of therapy among those found through the alternative screening approaches. We then calculated the number needed to screen and treat (NNST) to prevent the incidence of one complication, since this is the ultimate goal of early detection and treatment of type 2 diabetes; understanding the magnitude of benefit in reduction of complication risk is an essential component of WHO screening criteria. We also examined the costs of screening, including programmatic costs. We quantified the degree of sensitivity and uncertainty in all of these calculations, identifying the extent to which information available in currently available datasets could inform policy decisions despite continued uncertainty about type 2 diabetes pathogenesis and prevalence in India.

## Methods

Ethics committee approval for the Indian Migration Study (IMS) that was used to inform the model was obtained from the All India Institute of Medical Sciences Ethics Committee, reference number A-60/4/8/2004; for the overall modeling research, ethics committee approval was obtained from the Stanford University Institutional Review Board, reference number eP-28811.

### Model Overview

**Fig 1:**
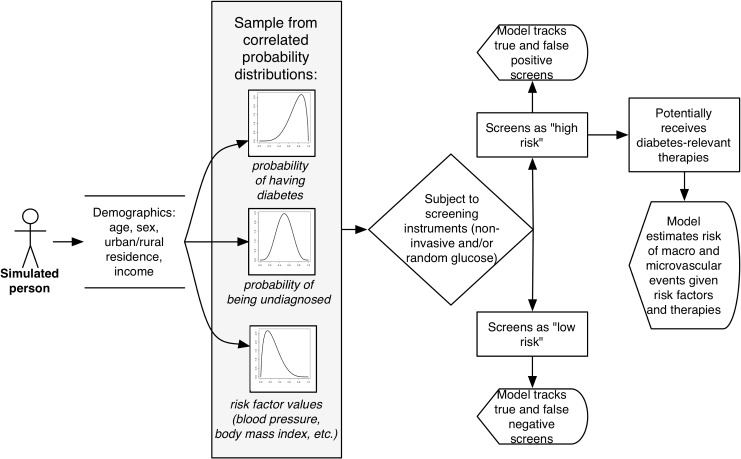
Model diagram. Individuals in the simulated population are assigned demographic characteristics based on the joint probabilities of being in each age, sex, location, and income group given population demographic estimates for India. They are then assigned a probability of having diabetes, either diagnosed or undiagnosed, and having various associated co-morbid risk factors, based on the joint probabilities of these prevalence rates and factors listed in S2 Table and illustrated in S1 Fig based on prior population estimates for India. Individuals are then subject to the screening instruments listed in Table 1, from which the model estimates positive and negative test results and subsequent diabetes complications with and without treatment.

The modeling proceeded in three stages (see Fig 1). Full details of the modeling process, including all input parameters, are provided in S1 Text, in accordance with international model reporting guidelines [16]. Here, we provide an overview of the input data and calculation approach.

**Table 1:** Alternative risk factors included in survey-based screening instruments proposed for detecting undiagnosed diabetes in India [6–8].

Elements Included	Risk Score Assigned in Each Instrument		
	**Chaturvedi Risk Score**	**Mohan Risk Score (Indian Diabetes Risk Score)**	**Ramachandran Risk Score**
Age	+0 points: <40 y;	+0 points: <35 y;	+0 points: <30 y;
	+4 points: 40–49 y;	+20 points: 35–49 y;	+10 points: 30–44 y;
	+6 points: >49 y	+30 points: ≥50 y	+18 points: 45–59 y;
			+19 points: >59 y
Blood pressure	+0 points: <120 mm Hg systolic and <80 mm Hg diastolic;	Not included	Not included
	+5 points: 120–139 mm Hg systolic or 80–89 mm Hg diastolic;		
	+7 points: ≥140 mm Hg systolic or ≥90 mm Hg diastolic		
Body mass index (BMI)	Not included	Not included	+0 points: BMI < 25 kg/m^2^;
			+7 points: BMI ≥ 25 kg/m^2^
Family history of diabetes	+0 points: no history;	+0 points: no history;	+0 points: no history;
	+4 points: history in parents or siblings	+10 points: either parent with history of diabetes;	+7 points: family history (unspecified members)
		+20 points: both parents with history of diabetes	
Physical activity level	Not included	+0 points: regular exercise and strenuous work;	+0 points: moderate or intense activity;
		+20 points: regular exercise or strenuous work;	+4 points: sedentary, light physical activity only
		+30 points: no exercise and no strenuous work	
Waist circumference	+0 points: ≤75 cm (female), ≤80 cm (male);	+0 points: <80 cm (female), <90 cm (male);	+0 points: <80 cm (female), <85 cm (male);
	+9 points: 76–84 cm (female), 81–89 cm (male);	+10 points: 80–89 cm (female), 90–99 cm (male);	+5 points: ≥80 cm (female), ≥85 cm (male)
	+12 points: >85 cm (female), >90 cm (male)	+20 points: ≥90 cm (female), ≥100 cm (male)	
Total risk score possible	29	100	42
Score considered “positive” for risk of undiagnosed diabetes, based on receiver operating characteristic (ROC) curve	16	60	21
Criterion for diabetes diagnosis	Fasting plasma glucose ≥ 7.0 mmol/l	2-h plasma glucose ≥ 11.1 mmol/l	2-h glucose (blood/plasma) ≥ 11.1 mmol/l

The model subjects each simulated individual to each of the listed screening instruments to identify how many people would test positive or negative by each instrument.

First, we constructed a synthetic, nationally representative Indian population from a series of sub-national cohort studies. We estimated the probability of having diabetes, the probability of being diagnosed, and the correlated distribution between these probabilities and key diabetes risk factors and co-morbidities (see Table 1 and S1 Fig) by age, sex, location, and income, using mixed-effects meta-regression.

Second, we subjected each member of the synthetic population to each of three survey-based screening instruments advocated for the detection of people with undiagnosed diabetes in India (see Table 1) [6–8], implemented as an alternative to or in combination with random glucose testing [15]. We tracked the number of people with previously undiagnosed diabetes who would be detected through screening (true positives) or would be missed (false negatives), and the number of people without diabetes who would be false positives or true negatives upon screening, given the distribution of risk factors among these groups and the different risk factors incorporated into each instrument (Table 1).

### Disease Burden

To estimate the burden of diabetes in India, we constructed a synthetic, nationally representative population for our model. We used United Nations estimates of India’s demographics for the year 2015 [17], dividing a simulated population of India into cohorts defined by age (25–44 y old and 45–65 y old—the age groups among whom screening efforts are focused and risk equations for complications have been validated [18,19]), sex, location (urban or rural), and income (tertiles of the standard of living index, the standard metric of wealth in India [20]). The lower-bound age cutoff was chosen based on the extremely small prevalence rates of type 2 diabetes below age 25 y in India, such that screening performance always worsens when including persons below this age; the upper-bound age cutoff was chosen based on the cutoff commonly used in current screening efforts [10] and the absence of clinical trial data for the effects of glycemic control beyond this age [21].

We adopted a microsimulation approach, which means each member of the population was simulated as an individual, and assigned a probability of having diabetes, a probability of being undiagnosed if having diabetes, and a set of risk factor values and co-morbidities (itemized in Table 1) correlated to these two probabilities. The advantage of this technique over older Markov modeling approaches is that microsimulation can capture complex individual screening experiences and co-morbid disease histories, providing an estimate of heterogeneous effects in a population as opposed to a simple population average among a given cohort. To assign individuals a diabetes disease status, determine whether they were likely to have been diagnosed if they had diabetes, and provide them with associated risk factor values, we performed Monte Carlo sampling from joint probability distributions of each of these probabilities and risk factors specific to their age, sex, income, and location group, estimated from a meta-regression on 58 cohort studies of diabetes published in India (*n* = 447,481; S1 Table). The cohort studies describe the prevalence of previously diagnosed diabetes (via self-report), the prevalence of previously undiagnosed diabetes (established through testing of the cohort populations per WHO diagnostic criteria [22,23]), and the correlated frequency of the risk factors and co-morbid conditions listed in Table 1. The overall probability distributions for diabetes disease, diabetes diagnosis, and associated risk factors were estimated through mixed-effects meta-regression accounting for changes in both disease prevalence and the probability of diagnosis over time. This is a method to infer a population metric from a group of studies rather than a single cohort, accounting for variations in sampling among studies. Specifically, the model applied to the total diabetes prevalence, the probability of being previously undiagnosed, and the risk factor value/co-morbidity prevalence observations *y* among the *j* studies was specified as [24]

$$y_{j}=\beta_{0}+\beta_{1}x_{1j}+\ldots +\beta_{nj}x_{nj}+\beta_{y}\text{year}+u_{j}+\varepsilon_{j}$$ (1)

where *x*_1*i*_ denotes the value of the first moderator variable (e.g., percent of population in the first age category, 25–44 y old) in the *i*th study, and so on through all moderator classes of age, sex, income, and location, and *u*_*j*_ and ε_*j*_ are error terms referring to the amount of variability in the true value of the outcome that is not accounted for by the moderators included in the model and sample variances. The model was fitted with weights using a standard “inverse-variance” method, with Monte Carlo sampling of estimates specific to the age, sex, income, and location groups drawn from the probability distributions of the β coefficients in the fitted model. Model details and outputs are provided in S1 Text and S2 Table. To assess the face validity of our estimates, we compared them against three independent estimates from the Global Burden of Metabolic Risk Factors of Chronic Diseases Collaborating Group [25], the WHO Study on Global Ageing and Adult Health [26], and the International Diabetes Federation Diabetes Atlas [4].

### Screening Simulations

By applying each of the three screening instruments shown in Table 1 to the synthetic population, we estimated how each screening instrument would perform in terms of sensitivity, specificity, positive predictive value, and negative predictive value. We then calculated the number needed to screen (NNS) to detect one previously undiagnosed diabetes case, and computed the overall number of persons deemed high risk, who would be referred for confirmatory testing in both urban and rural locations, thereby providing an estimate of the burden of screening on the healthcare system. Note that waist circumference is commonly used in the screening instruments, given its superior performance compared to BMI in predicting type 2 diabetes risk among South Asians [27–29]; however, BMI is included in one screening instrument, adopting a lower cut point than the international obesity cut point, given that diabetes risk in South Asians increases at a lower BMI, according to prior analyses [30,31].

The survey-based screening instruments listed in Table 1 were also compared to random glucometer testing, for which a previous trial in rural India found that the optimal threshold for screening by ROC curve analysis was ≥6.1 mmol/l [15]. We estimated how many people would exceed this threshold, incorporating the variation in blood glucose levels among both persons with undiagnosed diabetes and persons without diabetes (S1 Fig) and the degree to which the measures of random glucose via handheld glucometers in the Indian field trial differed from laboratory venous blood glucose test results (a standard deviation of 0.9 in Bland-Altman analysis) [15]. In all cases, the screening instruments were followed by laboratory-based fasting blood glucose testing for diagnosis because other methods (i.e., hemoglobin A1c) are not widely available in India or other low- and middle-income countries at present. We additionally determined what proportion of screened patients would be expected to be positive for additional co-morbidities such as hypertension and obesity by noting the prevalence of these conditions among the screen-positive patients (based on the joint distributions of co-morbidities illustrated in S1 Fig). We also combined the survey-based instruments and random glucometer testing to evaluate the sensitivity and specificity of both serial and parallel screening scenarios, specifically simulating every possible combination of screening questionnaires and random glucose testing in which either one test is followed by another in any order (and both tests need to be positive for referral to confirmatory testing) or both tests are performed simultaneously (and either test needs to be positive to proceed to confirmatory testing). We applied the instruments selectively to sub-populations defined by age, sex, urban/rural location, and income status to examine variations in sensitivity and specificity.

### Cost Analysis

To provide a sense of the typical costs associated with a screening program, we tabulated the costs of both questionnaire-based and glucometer-based screening. We used World Health Organization CHOICE (CHOosing Interventions that are Cost-Effective) data [32], which provide the unit costs for personnel, operations, and materials for screening (itemized in S3 Table). Total costs including overhead expenditures were tabulated in 2014 US dollars for each screening strategy over a 10-y implementation horizon at a standard 3% annual discount rate, and were subjected to the sensitivity and uncertainty analyses described below.

### Diabetes Complication Rates

To estimate the health benefits of screening, we used a mathematical model, the United Kingdom Prospective Diabetes Study (UKPDS) Outcomes Model 2, which includes South Asian–specific disease progression parameters that have been validated among South Asians in both the UK and India [33,34]. The risk equations in this model provide estimates of disease progression rates with and without treatment, including 20-y probabilities of heart disease events (including ischemic heart disease, myocardial infarction, and heart failure), stroke, blindness, diabetic ulcer (from peripheral arterial disease or neuropathy), and renal failure (see S1 Text for endpoint definitions) [18,19,33]. We drew relevant biomarker values for individuals found through screening using Monte Carlo sampling from the distribution of biomarkers among people with previously undiagnosed diabetes in the IMS, in which the biomarkers were assessed across India by age, sex, income, and location (see S1 Fig) [35]. We then estimated the minimum NNST to prevent each of the diabetes complications, using the most optimistic and aggressive targets for treatment (those set by the American Diabetes Association) to provide a minimum floor estimate of the NNST [21]. These targets include lowering systolic blood pressure to ≤140 mm Hg, low-density lipoprotein (LDL) to ≤2.6 mmol/l in those with no cardiovascular disease history (or ≤1.8 mmol/l in those with a history of cardiovascular disease), and hemoglobin A1c to ≤7%. We additionally simulated the observed impact of behavioral risk factor modification (i.e., lifestyle interventions) to reduce diabetes complications; although these have not been found effective for cardiovascular endpoints, they potentially reduce the risk of intermediate microvascular endpoints, which may translate into eventual reductions in blindness and end-stage renal failure [36,37]. To estimate the minimum NNST values (i.e., the most optimistic case), we simulated full reversal of risk, as per the UKPDS risk equations, and simulated the case of universal treatment adherence by both providers and patients. This approach provides the best-case scenario for treatment outcomes. We also simulated treatment of currently diagnosed patients with diabetes, similarly assigned biomarkers through Monte Carlo sampling from their distribution of risk factors in the IMS [35]. We validated our estimates against previously published example calculations to ensure correct application of the model equations [18,19,33].

### Sensitivity and Uncertainty Analyses

First, we estimated the minimum NNS to find one previously undiagnosed diabetes case if people eligible for screening were subjected directly to fasting venous blood glucose testing rather than the survey-based screening or random glucometer screening.

Second, we determined whether alternative thresholds for classifying persons as high risk for each instrument would maximize the performance (i.e., increase the product of the sensitivity and specificity) of the instrument among the national population in the model, as compared to the existing instrument cut points published previously based on sub-national studies (Table 1). We constructed ROC curves on all tested instruments to identify optimal cut points in the national population.

Finally, for uncertainty analysis, we ran our model 10,000 times while using Monte Carlo sampling from the probability distributions of all input parameters to estimate stable 95% credible interval estimates around all model outcomes.

The model was programmed and implemented in *R* (v. 3.1.0, The R Foundation for Statistical Computing, Vienna).

## Results

### Disease Burden

Among the 586 million people anticipated to be aged 25–65 y old in India in the year 2015, the mean diabetes prevalence rate was estimated as 12.0% in our model (95% CI: 8.4%–15.6%), such that an estimated 70 million (95% CI: 50 million–91 million) people in this age group are thought to have diabetes. In the simulated population, diabetes prevalence was slightly but not significantly higher among men (12.3%, 95% CI: 9.4%–15.2%) than women (11.7%, 95% CI: 7.5%–16.0%), and higher among urban (18.6%, 95% CI: 13.1%–24.1%) than rural (8.7%, 95% CI: 6.1%–11.2%) populations. An estimated 51 million of the 70 million persons with diabetes are expected to be undiagnosed before the screening program (73.3%, 95% CI: 69.9%–76.7%), with particularly high probabilities of being undiagnosed among women (83.1%, 95% CI: 81.3%–84.9%), low-income individuals (86.3%, 95% CI: 83.3%–89.3%), and rural populations (80.0%, 95% CI: 77.2%–82.8%). Fully disaggregated estimates by demographic group are provided in S2 Table.

We compared our modeled estimates of diagnosed and total diabetes prevalence to three independent estimates [4,25,26]. As shown in S4 Table, the model closely matched the independent estimates (<1.6% absolute difference) among all demographic categories and across all years for which estimates are available from 2000 through 2014.

### Screening Simulation

Table 2 summarizes the estimated sensitivity, specificity, positive predictive value, and negative predictive value of each instrument, as well as the estimated NNS to find one undiagnosed diabetes case. The Chaturvedi risk score had the highest sensitivity (72.8%, versus 50.8% and 64.9% for the other two risk scores) and intermediate specificity (58.0%, versus 64.0% and 47.1%), and has the lowest NNS to find one undiagnosed diabetes case (15.2, versus 21.7 and 17.0).

**Table 2:** Comparison of instrument performance in published sub-national populations versus synthetic national population [6–8,15].

Performance Category	Performance of Instrument in Detecting Undiagnosed Diabetes							
	**Chaturvedi Risk Score**		**Mohan Risk Score (Indian Diabetes Risk Score)**		**Ramachandran Risk Score**		**Random Point-of-Care Glucose Testing (≥6.1 mmol/l)**	
	**Published Estimates from Sub-National Cohorts**	**Estimate from Model (Synthetic National Cohort)**	**Published Estimates from Sub-National Cohorts**	**Estimate from Model (Synthetic National Cohort)**	**Published Estimates from Sub-National Cohorts**	**Estimate from Model (Synthetic National Cohort)**	**Published Estimates from Sub-National Cohorts**	**Estimate from Model (Synthetic National Cohort)**
Sensitivity	73% (68%–77%) in industrial workforce cohort from multiple Indian sites, 2001–2003; 66% (95% CI: 59%–73%) in urban Delhi and rural Haryana, 1991–1994	72.8 (71.5–74.1)	73% in urban and rural Chennai, 2001–2002 (no credible intervals reported)	50.8 (49.9–51.8)	77% and 72% in two cohorts from six cities (2000); 74% in a cohort from Chennai (1995); 92% in the South Asian cohort from the Health Survey for England (1999) (no credible intervals reported)	64.9 (63.9–65.9)	78% in rural Andhra Pradesh (no credible intervals reported)	62.6 (61.1–64.2)
Specificity	56% (55%–57%) in industrial workforce cohort from multiple Indian sites, 2001–2003; 67% (95% CI: 65%–68%) in urban Delhi and rural Haryana, 1991–1994	58.0 (57.5–58.5)	60% in urban and rural Chennai, 2001–2002 (no credible intervals reported)	64.0 (63.5–64.5)	60% and 59% in two cohorts from six cities (2000); 61% in a cohort from Chennai (1995); 26% in the South Asian cohort from the Health Survey for England (1999) (no credible intervals reported)	47.1 (46.8–47.4)	79% in rural Andhra Pradesh (no credible intervals reported)	75.5 (75.2–75.8)
Positive predictive value	6% (5%–7%) in industrial workforce cohort from multiple Indian sites, 2001–2003; 10% (8%–12%) in urban Delhi and rural Haryana, 1991–1994	14.7 (6.1–23.2)	17% in urban and rural Chennai, 2001–2002 (no credible intervals reported)	11.1 (3.8–18.5)	9% and 8% in two cohorts from six cities (2000); 12% in a cohort from Chennai (1995); 22% in the South Asian cohort from the Health Survey for England (1999) (no credible intervals reported)	9.9 (3.2–16.5)	15% in rural Andhra Pradesh (no credible intervals reported)	18.4 (7.5–29.2)
Negative predictive value	98% (97%–99%) in industrial workforce cohort from multiple Indian sites, 2001–2003; 97% (96%–98%) in urban Delhi and rural Haryana, 1991–1994	95.5 (92.6–98.4)	95% in urban and rural Chennai, 2001–2002 (no credible intervals reported)	93.3 (88.8–97.8)	98% and 98% in two cohorts from six cities (2000); 97% in a cohort from Chennai (1995); 94% in the South Asian cohort from the Health Survey for England (1999) (no credible intervals reported)	93.5 (89.1–97.8)	99% in rural Andhra Pradesh (no credible intervals reported)	95.5 (92.5–98.6)
NNS to detect one previously undiagnosed person with diabetes	Not reported	15.2 (6.0–23.5)	Not reported	21.7 (9.9–33.6)	Not reported	17.0 (7.7–26.3)	Not reported	17.6 (8.0–27.3)

95% credible intervals are shown in parentheses. In all cases, the screening instrument is the first-stage test, and individuals testing positive are then subject to fasting blood glucose testing for diagnostic confirmation.

By comparison with these survey-based screening instruments, random glucometer testing offered greater specificity and therefore fewer false-positive results (see Table 2). Random glucometer testing would be expected to have 62.6% sensitivity (95% CI: 61.1%–64.2%) and 75.5% specificity (95% CI: 75.2%–75.8%), requiring 17.6 persons (95% CI: 8.0–27.3) to be screened to find one undiagnosed diabetes case.

**Fig 2:**
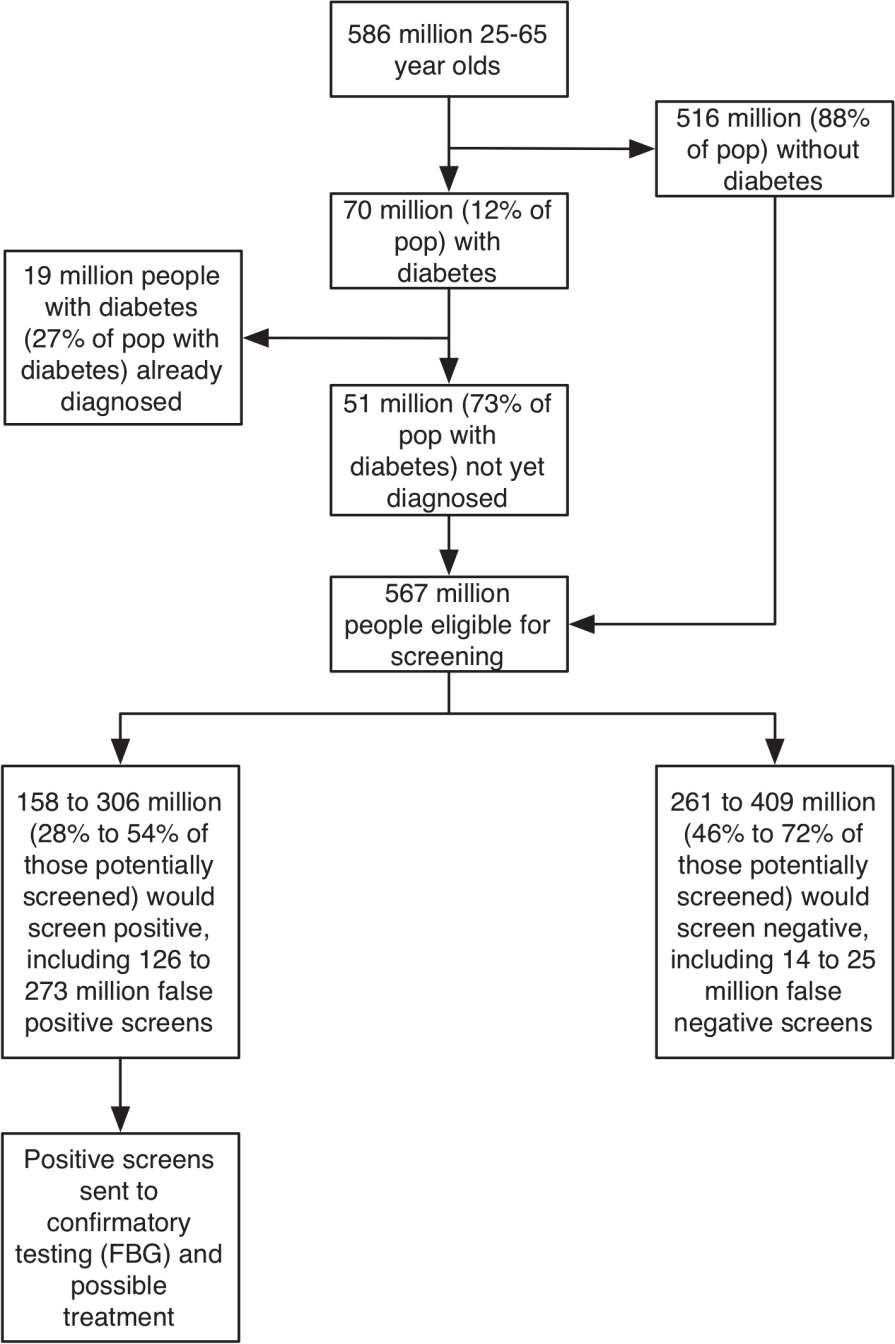
Population-level implications of large-scale screening for diabetes in India. FBG, fasting blood glucose.

When projected to the population level (Fig 2; Table 3), we estimated that among 567 million Indians aged 25–65 y eligible for screening, between 158 and 306 million people (27.9%–53.9%) would screen positive and need referral for confirmatory testing. Between 26 and 37 million of the 51 million people with undiagnosed diabetes would be detected through any of the screening instruments (50.9%–72.8% of the undiagnosed population), while between 14 and 25 million people with undiagnosed diabetes would be missed by the screening instruments (27.2%–49.1% of those undiagnosed). Because of the lack of specificity of the survey-based screening instruments, between 186 and 273 million people without diabetes (36.0%–52.8% of those without diabetes screened) would be identified as false positives after confirmatory testing. For comparison, using a coin flip to refer people for confirmatory testing would detect 50% of undiagnosed diabetes (25.5 million people) and refer about 258 million people without diabetes to confirmatory testing. In the case of using random glucometer testing as an alternative screening approach, the number of false-positive screens would be expected to be reduced to 126 million (24.5% of those without diabetes screened). In other words, the ratio of false to true positives would range from 3.9 (in the case of random glucometer testing) to 8.2 (in the case of the Ramachandran risk score). S5 Table provides a complete sensitivity analysis of screening outcomes if screening were targeted to certain demographic groups and locations, revealing a consistently high ratio of false to true positives across targeted screening approaches.

**Table 3:** Health system burden associated with alternative diabetes screening approaches.

Population or Cost	Burden by Instrument			
	Chaturvedi Risk Score	Mohan Risk Score (Indian Diabetes Risk Score)	Ramachandran Risk Score	Random Point-of-Care Glucose Testing (≥6.1 mmol/l)
**People with undiagnosed diabetes (millions)**				
True-positive screens (percent of people with undiagnosed diabetes screening positive)	37.3 (37.3–37.3) (72.8% of undiagnosed)	26.1 (26.0–26.1) (50.9% of undiagnosed)	33.2 (33.2–33.3) (64.9% of undiagnosed)	32.1 (32.1–32.1) (62.7% of undiagnosed)
False-negative screens (percent of people with undiagnosed diabetes screening negative)	13.9 (13.9–13.9) (27.2% of undiagnosed)	25.1 (25.1–25.2) (49.1% of undiagnosed)	18.0 (18.0–18.0) (35.1% of undiagnosed)	19.1 (19.1–19.1) (37.3% of undiagnosed)
**People without diabetes eligible for screening (having previously unknown diabetes status) (millions)**				
True-negative screens (percent of people without diabetes screening negative)	299.4 (299.4–299.5) (58.1% of people without diabetes screened)	330.2 (330.2–330.2) (64% of people without diabetes screened)	243.1 (243.1–243.2) (47.2% of people without diabetes screened)	389.5 (389.5–389.5) (75.5% of people without diabetes screened)
False-positive screens (percent of people without diabetes screening positive)	216.2 (216.2–216.2) (41.9% of people without diabetes screened)	185.5 (185.5–185.5) (36% of people without diabetes screened)	272.5 (272.5–272.5) (52.8% of people without diabetes screened)	126.2 (126.2–126.2) (24.5% of people without diabetes screened)
**Total positive screens (percent of those screened being referred to confirmatory testing) (millions)**	253.5 (253.5–253.5) (44.7% of those screened)	211.6 (211.6–211.6) (37.3% of those screened)	305.8 (305.8–305.8) (53.9% of those screened)	158.3 (158.3–158.3) (27.9% of those screened)
**Estimated cost of implementation including screening and confirmatory testing costs (millions of 2014 US dollars)**	$484.99 ($341.80–$632.54)	$397.31 ($279.37–$517.13)	$567.17 ($398.26–$737.66)	$169.48 ($119.90–$221.34)
**Estimated screening and confirmatory testing cost per case (true-positive screen) found (2014 US dollars)**	$13.01 ($9.17–$16.96)	$15.25 ($10.73–$19.84)	$17.06 ($11.98–$22.18)	$5.28 ($3.74–$6.90)

The total number of people eligible for screening across instruments is 566.876 million (95% CI: 560.954–572.797 million). 95% credible intervals are shown in parentheses. The table provides modeled estimates of how a simulated national population would be treated if screened through each instrument detailed in Table 1. In each case, the number of false-positive results is very large.

Of note, the diabetes screening strategies would also serve to detect co-morbid diseases (S1 Fig). Of the proportion of persons screening positive through each instrument, between 23% (Mohan risk score) and 79% (random glucometer testing) would also be expected to have hypertension, and between 13% (Mohan risk score) and 56% (random glucometer testing) would also be expected to meet the criterion for obesity (BMI ≥ 30 kg/m^2^).

**Fig 3:**
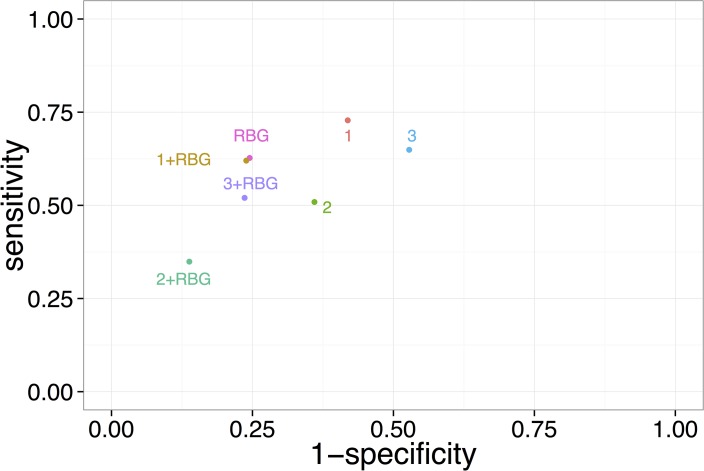
Comparison of each instrument in isolation, or when followed by random blood glucose testing (point-of-care capillary blood glucometer test). Numbers refer to screening instruments: 1, Chaturvedi risk score; 2, Mohan risk score; 3, Ramachandran risk score; RBG refers to random blood glucose testing. This plot displays the instrument performance using the cut points for positivity that were published in the literature previously to maximize the area under the ROC curve upon testing of the instruments among sub-national populations [6–8,15]. We also compared the performance of the instruments when recalibrated to the synthetic nationally representative population (S2 Fig).

We compared both serial and parallel screening scenarios, that is, when one instrument would be followed by another in any order (and both tests would need to be positive for referral to confirmatory testing) or both tests would be performed simultaneously (and either test would need to be positive for referral to confirmatory testing), respectively. All of the parallel screening scenarios had worse screening performance than the serial testing scenarios, because of extremely high false- to true-positive ratios (S6 Table). Performing serial testing with survey-based instruments after random glucometer testing also failed to improve screening performance. The best screening performance, by contrast, was achieved through serial testing when survey-based instruments were used first, followed by random glucose testing of a blood sample with a handheld glucometer. This did not produce a significant improvement in sensitivity over and above using random glucose testing alone, but the specificity of the screening could be improved to as much as 86% (reducing the number of false positives to 30 million, but not significantly lowering the false- to true-positive ratio; S6 Table). Fig 3 illustrates the ROC points for each screening instrument in isolation and when followed by random glucose testing.

### Cost Analysis

Table 3 summarizes the overall costs of each screening strategy, including estimated personnel, operations, and material costs anticipated from each screening strategy. We found that although the screening questionnaire costs were smaller than the costs of glucometer testing, this cost differential is more than outweighed by the high cost of confirmatory testing for the larger number of people who falsely screen positive by the questionnaire-based instruments. Overall discounted costs for screening varied from a low-end estimate of US$169 million for random glucometer testing (95% CI: US$120–US$221 million) to a high-end estimate of US$567 million for the Ramachandran risk score (95% CI: US$398–US$737 million), which corresponds to a price per case found of between US$5.28 (95% CI: US$3.74–US$6.90) for random glucometer testing and US$17.06 (95% CI: US$11.98–US$22.18) for the Ramachandran risk score.

### Diabetes Complication Rates

In order to provide a sense of the order-of-magnitude difference in potential benefits of treatment among those with diabetes newly detected through screening versus those already diagnosed, S7 Table summarizes the model-based estimates of complication rates among the newly screened population. As shown in the table, in the best-case treatment scenario the NNST among those diagnosed through the community-based screening program was very high: on the order of 789 people (95% CI: 225–2,213). Tallying the individual complications averted through therapy, we found that 6.8% of the population newly diagnosed due to the screening program (95% CI: 2.3%-28.6%) would be expected to avoid a diabetes complication over 20 y in this best-case scenario. This corresponds to a median screening cost per case with complication prevented (i.e., excluding treatment costs) of US$1,908.17 (95% CI: US$562.00–US$7,015.81) in the best-case scenario.

### Sensitivity and Uncertainty Analyses

We also estimated the minimum NNS to find one previously undiagnosed diabetes case if people eligible for screening were subjected to fasting venous blood glucose testing, bypassing the survey-based screening instruments or random glucometer screening to skip directly to the confirmatory testing phase. The NNS through this approach to find one previously undiagnosed diabetes case was estimated to be 11.1 (95% CI: 8.6–15.6), as compared to between 15.2 and 21.7 for the alternative screening instruments (Table 2).

We found that recalibrating each screening instrument to the synthetic nationally representative population (S2 Fig), rather than using the standard cut points established in the sub-national cohort studies (Table 1), would allow us to improve the specificity of some of the instruments (S8 Table). Here, the recalibration involved choosing the cut point that maximized the product of sensitivity and specificity (i.e., the point at the top- and left-most corner of the ROC curves). This recalibration did not significantly change the NNS (Table 2, cf. S8 Table), and the number of false positives would still remain between 40 million and 116 million, for a false- to true-positive ratio of between 1.2 and 4.4.

## Discussion

Our study finds that large-scale, population-wide screening for type 2 diabetes among adults in India is unlikely to meet established criteria for implementing screening programs, which specify that such screening should utilize a reliably sensitive and specific instrument, and offer significant therapeutic benefits to those individuals diagnosed through screening [11].

Our investigation adds several important findings to the existing literature. First, we found that not all instruments tested previously in nonrepresentative subpopulations would be expected to work well in a nationally standardized large-scale screening program. For instance, the popular Indian Diabetes Risk Score (the Mohan risk score) was found to have a sensitivity no better than chance on average when extended to population-representative data, as opposed to the data from the single city in which it was previously evaluated [8]. Recalibrating the instruments to a nationally representative population did not significantly impact the NNS to detect one undiagnosed diabetes case. Second, among the instruments and approaches tested, we found that very large numbers of false-positive screens would be expected. At a minimum, 126 million people who do not have diabetes would be labeled as high risk and referred for confirmatory testing, and as many as 273 million false positives would be expected to be referred if applying some currently proposed screening instruments. When including both false- and true-positive screens, the burden on the health system would be expected to range from 158 to 306 million people referred to confirmatory testing, in order to detect an estimated 26 to 37 million people with currently undiagnosed diabetes. Third, among the screening options available, skipping the survey-based or random glucometer testing to proceed directly to fasting venous blood glucose appeared to significantly lower the NNS to find one previously undiagnosed diabetes case, from between 15 and 22 persons down to 11 persons. It may be argued that adopting such a measure requires laboratory infrastructure, and thereby limits the population reached through screening; conversely, the question remains how meaningful and safe glucose-lowering therapy would be delivered to those individuals newly diagnosed in settings without such capacity, given that screening processes must follow basic principles of non-maleficence (i.e., “first do no harm”) [11].

The microsimulation results imply that the benefits of population-based diabetes screening may be just as limited in India as in higher-income countries, where such screening has been shown not to produce a significant reduction in all-cause, cardiovascular, or diabetes-related mortality, or in rates of end-stage diabetes-related microvascular compilations such as blindness or renal failure. The studies in higher-income countries have involved cardiovascular risk reduction measures among screen-detected diabetes patients, and target-based treatment among diabetes patients detected through screening in high-risk populations [13,14]. In countries such as India where the burden of disease is rising in the context of healthcare system capacity limitations, a large-scale screening program bears the burden of demonstrating that the system can handle the effort required to provide confirmatory testing and significantly beneficial treatment among those screening positive and testing positive, respectively. Our assessment calls into question the ability for this to be achieved in India, given the poor performance of current screening instruments and the modest impact of early therapy for diabetes.

There are several strengths and some limitations of the present study. We conducted a comprehensive analysis that incorporates the most optimistic screening scenarios and iteratively varies screening instruments across all plausible approaches. We additionally sampled among diverse Indian populations to construct a nationally representative population for study. Yet, as with all assessments of potential public health interventions, our model is based on important assumptions. First, we used existing published cohort studies of diabetes prevalence. While our assessment generated diabetes prevalence estimates matching independent estimates, the cohort studies are still unlikely fully to capture the poorest and most rural areas of the country, which are thought to have the lowest diabetes prevalence. This limitation would tend to overstate the positive impacts of screening in our model. We additionally derived estimates of treatment effects based on a model primarily developed among South Asians in the UK, and using effect size estimates largely derived from clinical trials in higher-income populations. Further, to provide best-case estimates, we assumed that aggressive treatment goals (systolic blood pressure < 140 mm Hg, hemoglobin A1c < 7%, and LDL < 100 mg/dl) would be reached, though these goals are rarely achieved even in developed countries and are clearly not reflective of current treatment patterns in India [38]. We performed these calculations to provide relative magnitude estimates of treatment impact among newly screened versus previously diagnosed patients, not to provide precise forecasts for cost-effectiveness analysis. Furthermore, we focused exclusively on type 2 diabetes, which is believed to comprise the vast majority of diabetes cases in India; the screening approaches here are not valid for type 1 diabetes, which would typically manifest among much younger age groups. Finally, we attached cost estimates based on currently available data on cost, but cost variations over space and time would be expected to generate differences between expected and realized costs in practice.

Our findings have several implications for further research. In particular, we did not compare alternative guidelines that are being advocated for treatment strategies among those newly diagnosed with diabetes. The American Diabetes Association, World Health Organization, and International Diabetes Federation guidelines all slightly differ in their suggested management strategies for diabetes among adults, and would likely have different implications for patient risk, safety, and system-level resource requirements [21,39,40]. A formal comparative effectiveness assessment to tabulate the risks and benefits of each guideline-based treatment strategy is needed. Furthermore, we found that a significant number of persons screening as high risk for diabetes would also likely screen positive for untreated hypertension. While the mass screening for asymptomatic diabetes may not produce significant treatment benefits given the limited impact of early glycemic control for asymptomatic diabetes, further evaluation of mass screening approaches for hypertension, with secondary screening of hypertensive individuals for diabetes risk, may be a prudent approach. This is particularly important given that the major cause of morbidity and mortality among people with type 2 diabetes in India and other low- and middle-income countries is cardiovascular disease, and the major all-cause mortality risk reduction benefit from diabetes treatment is from antihypertensive therapy rather than glycemic control [41,42]. An alternative to disease-specific screening may be an approach that focuses not on detection of individual risk factors, but on global cardiovascular risk assessment, based on a constellation of observed co-morbid risk factors ranging from diabetes to hypertension to tobacco smoking [43,44]. However, the benefit of cardiovascular risk assessment programs has been questioned, and their feasibility and cost-effectiveness in low- and middle-income countries need to be carefully considered [45].

Despite the need for future research as suggested above, our current analysis suggests that the existing and expanding large-scale diabetes screening program in India, which parallels the programs being suggested for implementation in numerous low- and middle-income countries [5,46], may have difficulties fulfilling basic criteria considered essential for effective large-scale screening. The current analysis suggests that no population-based mass diabetes screening option can truly be recommended at present because of the vast expected number of false-positive results. Hence, given our results, an approach that focuses on symptom-based screening, with attendant treatment improvement among already-diagnosed persons, may be more sensible than community-based mass screening. Improving instruments to reduce false-positive screens, preparing the health system for very substantial confirmatory testing demands, and identifying how to deliver efficacious treatment, are three priority areas that require urgent attention before countries experiencing rapid increases in diabetes prevalence implement large-scale community-based diabetes screening programs.

## Supporting Information

S1 Fig Joint probability distributions of diabetes and associated risk factors and co-morbidities. (DOCX)

S2 Fig Receiver operating characteristic curves for each survey-based screening instrument and random glucometer testing, applied to the synthetic nationally representative population to perform recalibration . Chaturvedi risk score, orange; Mohan risk score, gray; Ramachandran risk score, green; random glucometer testing, blue.
(DOCX)

S1 Table Indian cohort studies incorporated into the construction of the synthetic population of individuals with diagnosed and undiagnosed diabetes in India. (DOCX)

S2 Table Probability of diabetes and undiagnosed diabetes by demographic group. Estimates are for the year 2015. 95% credible intervals are shown in parentheses.
(DOCX)

S3 Table Unit component expenditures included in the cost analysis. (DOCX)

S4 Table Comparison of model estimates to three independent estimates for all available demographic groups and years of data. (DOCX)

S5 Table Sensitivity analysis of large-scale diabetes screening, targeted to different demographic groups. (DOCX)

S6 Table Combined impact of survey-based screening instruments with random glucometer testing. (DOCX)

S7 Table Diabetes complication rates before and after treatment. (DOCX)

S8 Table Performance characteristics of each instrument following recalibration to the synthetic nationally representative population. (DOCX)

S1 Text Detailed description of the modeling approach. (DOCX)

## Abbreviations:

BMI: body mass index
IMS: Indian Migration Study
LDL: low-density lipoprotein
NNS: number needed to screen
NNST: number needed to screen and treat
ROC: receiver operating characteristic
UKPDS: United Kingdom Prospective Diabetes Study
