# Supplementary material for: The Health System and Population Health Implications of Large-Scale Diabetes Screening in India: A Microsimulation Model of Alternative Approaches
Source: PLoS Med. 2015 May 19;12(5):e1001827. doi: 10.1371/journal.pmed.1001827 (PMC4437977; doi:10.1371/journal.pmed.1001827)

S1 Fig.: Joint probability distributions of diabetes and associated risk factors and co-morbidities. Samples from distributions of: (A) continuous risk factors, biomarkers and co-morbidities among previously-undiagnosed people with diabetes in India, including kernel density estimates along the diagonal and locally-weighted regression lines to illustrate nonlinearity of correlations (red curves with dashed 95% credible intervals, contrasted with green linear regressions); and frequency distributions of (B) categorical risk factors, biomarkers and co-morbidities among previously-undiagnosed people with diabetes in India, along with their 95% credible intervals.

(A) Legend (from top left to bottom right): Agedx = age at diagnosis (yrs), A1c = hemoglobin A1c (%), SBP = systolic blood pressure (mmHg), DBP = diastolic blood pressure (mmHg), LR = lipid ratio (total/HDL cholesterol), HR = heart rate (beats/min), WBC = white blood count (10^6/mL), BMI = body mass index (kg/m^2), Waist = waist circumference (cm), GFR = glomerular filtration rate (ml/min/1.73m^2), Hgb = hemoglobin (g %), LDL = low density lipoprotein cholesterol (mmol/L)


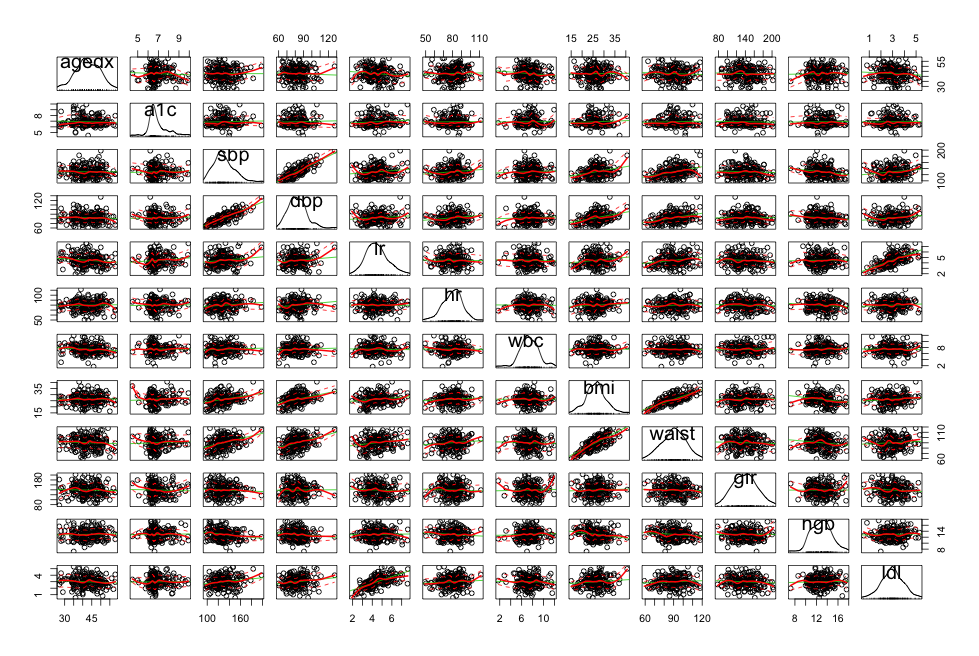


(B)

Legend: PVD: history of peripheral vascular disease; Smoking: tobacco smoking currently; Blind: history of blindness; Amp: history of amputation; IHD: history of ischemic heart disease; CHF: history of congestive heart failure; Microalb: microalbuminuria (urine albumin ≥50 mg/L); FH: family history; PA: physical activity.


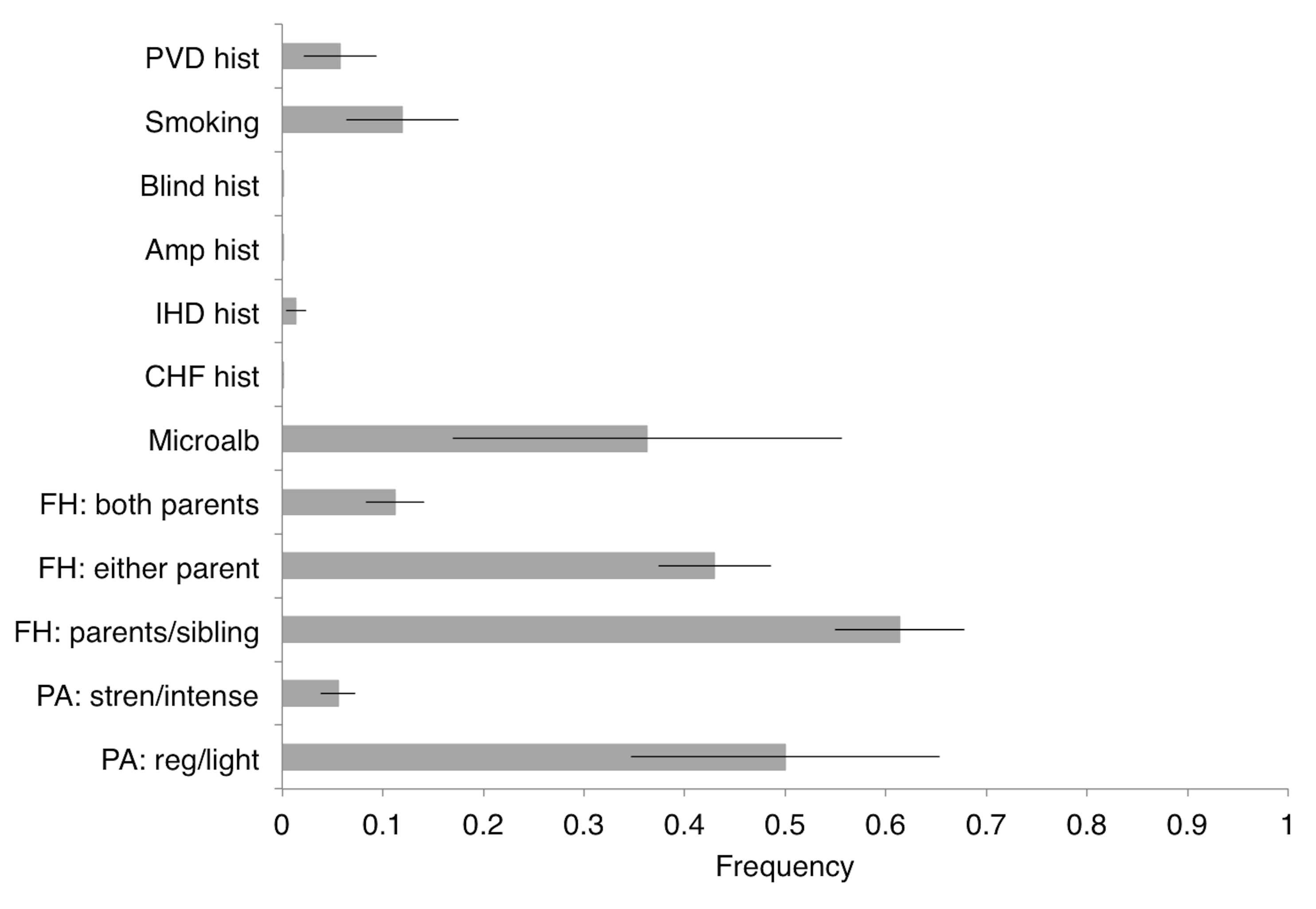

Supplement: S1 Fig — (DOCX) [file pmed.1001827.s001.docx]
