# Supplementary material for: The Health System and Population Health Implications of Large-Scale Diabetes Screening in India: A Microsimulation Model of Alternative Approaches
Source: PLoS Med. 2015 May 19;12(5):e1001827. doi: 10.1371/journal.pmed.1001827 (PMC4437977; doi:10.1371/journal.pmed.1001827)
Supplement: S2 Fig — . Chaturvedi risk score, orange; Mohan risk score, gray; Ramachandran risk score, green; random glucometer testing, blue. (DOCX) [file pmed.1001827.s002.docx]

S2 Fig.: Receiver operating characteristic curves for each survey-based screening instrument and random glucometer testing, applied to the synthetic nationally-representative population to perform recalibration. Instrument numbers and results of the recalibration are displayed in SI Table S8. 95% credible intervals are shown in parentheses.

Legend: Orange = instrument 1, Gray = instrument 2, Green = instrument 3, Blue = random glucometer testing. Lines = new recalibrated ROC curves; Squares = old cut-points (as in Figure 3).
