# Supplementary material for: The Health System and Population Health Implications of Large-Scale Diabetes Screening in India: A Microsimulation Model of Alternative Approaches
Source: PLoS Med. 2015 May 19;12(5):e1001827. doi: 10.1371/journal.pmed.1001827 (PMC4437977; doi:10.1371/journal.pmed.1001827)
Supplement: S1 Table — (DOCX) [file pmed.1001827.s003.docx]

S1 Table: Indian cohort studies incorporated into the construction of the synthetic population of diagnosed and undiagnosed diabetics in India. We combined estimates weighted by sample size from each of these studies to construct probability distributions of total and undiagnosed diabetes by age, sex, income, urban versus rural location, and year across India, and associated risk factor distributions relevant to diabetes risk instruments and complications (see S1 Figure). Where mid-year of prevalence was not clearly reported, the year prior to publication was used as listed below.

| *Cohort* | *Mid-year of diabetes prevalence rate estimate* | *N* |
| --- | --- | --- |
| Murthy PD, Pullaiah B, Rao KV. Survey for detection of hyperglycemia and diabetes mellitus in Tenali. In: Bajaj JS, editor. Diabetes mellitus in developing countries. New Delhi: Interprint; 1984. p. 55. | 1984 | 848 |
| Verma NPS, Mehta SP, Madhu SV, Mather HM, Keen H. Prevalence of known diabetes mellitus in an urban Indian environment: the Darya Ganji diabetes survey.Br Med J. 1986;293:423–4. | 1986 | 6,878 |
| Iyer SR, Chauhan RB, Khandwala RM, Shah PC. Diabetes mellitus in and around Bardoli. J Diabetic Assoc India. 1987;27:11–4. | 1987 | 1,348 |
| Ramachandran A, Jali MV, Mohan V, Snehalatha C, and Vishwanathan M: High prevalence of diabetes in an urban population in south India. Br Med J 1988; 297: 587. | 1988 | 678 |
| Bai PV, Krishnaswami CV, Chellamariappan M. Prevalence and incidence of type-2 diabetes and impaired glucose tolerance in a selected Indian urban population. J Assoc Physicians India. 1999 Nov;47(11):1060–4. | 1992 | 1,198 |
| Ramachandran A, Snehalatha C, Dharmaraj D, Viswanathan M. Prevalence of glucose intolerance in Asian Indians.Urban-rural difference and significance of upper body adiposity. Diabetes Care. 1992;15:1348–55 | 1992 | 1,938 |
| Patandin S, Bots ML, Abel R, Valkenburg HA. Impaired glucose tolerance and diabetes mellitus in a rural population in south India. Diabetes Res Clin Pract. 1994 May;24(1):47–53. | 1993 | 467 |
| Wander GS, Khurana SB, Gulati R, Sachar RK, Gupta RK, Khurana S, et al. Epidemiology of coronary heart disease in a rural Punjab population--prevalence and correlation with various risk factors. Indian Heart J. 1994 Dec;46(6):319–23. | 1994 | 1,100 |
| Ramachandran A, Snehalatha C, Latha E, Vijay V, Viswanathan M. Rising prevalence of NIDDM in an urban population in India. Diabetologia. 1997 Feb;40(2):232–7. | 1995 | 2,183 |
| Shah SK, Saikia M, Barman NN, Snehalatha C, Ramachandran A. High prevalence of type 2 diabetes in urban population in north-eastern. Int J Diab Developing Countries. 1998;18:97–101. | 1998 | 1,016 |
| Singh RB, Bajaj S, Niaz MA, Rastogi SS, Moshiri M. Prevalence of type 2 diabetes mellitus and risk of hypertension and coronary artery disease in rural and urban population with low rates of obesity. Int J Cardiol 1998; 66: 65-72. | 1998 | 3,575 |
| Kutty VR, Soman CR, Joseph A, Pisharody R, Vijayakumar K. Type 2 diabetes in southern Kerala: variation in prevalence among geographic divisions within a region. Natl Med J India. 2000 Dec;13(6):287–92. | 1999 | 3,899 |
| Qiao Q, Hu G, Tuomilehto J, Nakagami T, Balkau B, Borch-Johnsen K, et al. Age- and sex-specific prevalence of diabetes and impaired glucose regulation in 11 Asian cohorts. Diabetes Care 2003; 26: 1770-80. | 1999 | 9,848 |
| Sridhar GR, Putcha V, Lakshmi G. Time trends in the prevalence of diabetes mellitus: ten year analysis from southern India (1994-2004) on 19,072 subjects with diabetes. J Assoc Physicians India. 2010 May;58:290–4 | 1999 | 19,072 |
| Zargar AH, Khan AK, Masoodi SR, Laway BA, Wani AI, Bashir MI, et al. Prevalence of type 2 diabetes mellitus and impaired glucose tolerance in the Kashmir Valley of the Indian subcontinent. Diabetes Res Clin Pract. 2000 Feb;47(2):135–46. | 1999 | 5,083 |
| Mohan V, Shanthirani S, Deepa R, Premalatha G, Sastry NG, Saroja R, et al. Intra-urban differences in the prevalence of the metabolic syndrome in southern India -- the Chennai Urban Population Study (CUPS No. 4). Diabet Med. 2001 Apr;18(4):280–7. | 1999 | 1,262 |
| Ramachandran A, Snehalatha C, Vijay V. Temporal changes in prevalence of type 2 diabetes and impaired glucose tolerance in urban southern India. Diabetes Res Clin Pract 2002; 58: 55-60. | 1989,  1995,  2000 | 900,  2,183,  1,668 |
| Iyer SR, Iyer RR, Upasani SV, Baitule MN. Diabetes mellitus in Dombivli--an urban population study. J Assoc Physicians India. 2001 Jul;49:713–6. | 2000 | 520 |
| Misra A, Pandey RM, Devi JR, Sharma R, Vikram NK, Khanna N. High prevalence of diabetes, obesity and dyslipidaemia in urban slum population in northern India. Int J Obes Relat Metab Disord. 2001 Nov;25(11):1722–9. | 2000 | 532 |
| Ramachandran A, Snehalatha C, Kapur A, Vijay V, Mohan V, Das AK, et al. High prevalence of diabetes and impaired glucose tolerance in India: National Urban Diabetes Survey. Diabetologia 2001; 44: 1094-101. | 2000 | 11,216 |
| Gupta A, Gupta R, Sarna M, Rastogi S, Gupta VP, Kothari K. Prevalence of diabetes, impaired fasting glucose and insulin resistance syndrome in an urban Indian population. Diabetes Res Clin Pract 2003; 61: 69-76. 37. | 2001 | 1,091 |
| Sadikot SM, Nigam A, Das S, Bajaj S, Zargar AH, Prasannakumar KM, et al. The burden of diabetes and impaired glucose tolerance in India using the WHO 1999 criteria: prevalence of diabetes in India study (PODIS) Diabetes Res Clin Pract. 2004;66:301–7. | 2001 | 18,363 |
| Safraj S, Anish T, Vijayakumar K, Kutty VR, Soman CR. Socioeconomic position and prevalence of self-reported diabetes in rural Kerala, India: results from the PROLIFE study. Asia Pac J Public Health. 2012 May;24(3):480–6. | 2001 | 161,942 |
| Mohan V, Shanthirani CS, Deepa R. Glucose intolerance (diabetes and IGT) in a selected South Indian population with special reference to family history, obesity and lifestyle factors--the Chennai Urban Population Study (CUPS 14). J Assoc Physicians India. 2003 Aug;51:771–7. | 2002 | 1,262 |
| Gupta R, Sarna M, Thanvi J, Rastogi P, Kaul V, Gupta VP. High prevalence of multiple coronary risk factors in Punjabi Bhatia community: Jaipur Heart Watch-3. Indian Heart J 2004; 56: 646-52. | 2003 | 458 |
| Ramachandran A, Snehalatha C, Satyavani K, Vijay V. Impaired fasting glucose and impaired glucose tolerance in urban population in India. Diabet Med. 2003 Mar;20(3):220–4. | 2003 | 10,025 |
| Ramachandran A, Snehalatha C, Baskar ADS, Mary S, Kumar CKS, Selvam S, et al. Temporal changes in prevalence of diabetes and impaired glucose tolerance associated with lifestyle transition occurring in the rural population in India. Diabetologia. 2004 May;47(5):860–5. | 2003 | 1,213 |
| Mohan V, Deepa M, Deepa R, Shanthirani CS, Farooq S, Ganesan A, et al. Secular trends in the prevalence of diabetes and glucose tolerance in urban South India-the Chennai Urban Rural Epidemiology Study (CURES-17) Diabetologia.2006;49:1175–8. | 2003 | 2,350 |
| Fall CH, Sachdev HS, Osmond C, Lakshmy R, Biswas SD, Prabhakaran D, et al. Adult metabolic syndrome and impaired glucose tolerance are associated with different patterns of BMI gain during infancy: Data from the New Delhi Birth Cohort. Diabetes Care 2008; 31: 2349-56. | 2004 | 1,492 |
| Mohan V, Mathur P, Deepa R, Deepa M, Shukla DK, Menon GR, et al. Urban rural differences in prevalence of self-reported diabetes in India--the WHO-ICMR Indian NCD risk factor surveillance. Diabetes Res Clin Pract 2008; 80: 159-68. | 2004 | 44,523 |
| Prabhakaran D, Shah P, Chaturvedi V, Ramakrishnan L, Manhapra A, Reddy KS. Cardiovascular risk factor prevalence among men in a large industry of northern India. Natl Med J India. 2005 Apr;18(2):59–65. | 2004 | 2,122 |
| Sarkar S, Das M, Mukhopadhyay B, Chakrabarti CS, Majumder PP. High prevalence of metabolic syndrome and its correlates in two tribal populations of India and the impact of urbanization. Indian J Med Res. 2006 May;123(5):679–86. | 2005 | 563 |
| Chow CK, Raju PK, Raju R, Reddy KS, Cardona M, Celermajer DS, et al. The prevalence and management of diabetes in rural India. Diabetes Care.2006;29:1717–8. | 2006 | 4,535 |
| Ebrahim et al. The Effect of Rural-to-Urban Migration on Obesity and Diabetes in India: A Cross-Sectional Study. PLOS Medicine 2009. DOI: 10.1371/journal.pmed.1000268 | 2006 | 6,510 |
| Gupta R, Kaul V, Bhagat N, Agrawal M, Gupta VP, Misra A, et al. Trends in prevalence of coronary risk factors in an urban Indian population: Jaipur Heart Watch-4. Indian Heart J. 2007 Aug;59(4):346–53. | 2006 | 1,127 |
| Menon VU, Kumar KV, Gilchrist A, Sugathan TN, Sundaram KR, Nair V, et al. Prevalence of known and undetected diabetes and associated risk factors in central Kerala--ADEPS. Diabetes Res Clin Pract. 2006 Dec;74(3):289–94. | 2006 | 3,069 |
| Ramachandran A, Mary S, Yamuna A, Murugesan N, Snehalatha C. High prevalence of diabetes and cardiovascular risk factors associated with urbanization in India. Diabetes Care 2008; 31: 893-8. | 2006 | 7,066 |
| Ajay VS, Prabhakaran D, Jeemon P, Thankappan KR, Mohan V, Ramakrishnan L, et al. Prevalence and determinants of diabetes mellitus in the Indian industrial population. Diabet Med. 2008 Oct;25(10):1187–94. | 2007 | 10,930 |
| Bharati DR, Pal R, Kar S, Rekha R, Yamuna TV, Basu M. Prevalence and determinants of diabetes mellitus in Puducherry, South India. J Pharm Bioallied Sci. 2011 Oct;3(4):513–8. | 2007 | 1,370 |
| Vijayakumar G, Arun R, Kutty VR. High prevalence of type 2 diabetes mellitus and other metabolic disorders in rural Central Kerala. J Assoc Physicians India. 2009 Aug;57:563–7. | 2007 | 1,990 |
| Balagopal P, Kamalamma N, Patel TG, Misra R. A community-based participatory diabetes prevention and management intervention in rural India using community health workers. Diabetes Educ. 2012 Dec;38(6):822–34. | 2008 | 1,638 |
| Bharati DR, Pal R, Rekha R, Yamuna TV. Evaluation of the burden of type 2 diabetes mellitus in population of Puducherry, South India. Diabetes Metab Syndr. 2011   Mar;5(1):12–6. | 2008 | 1,013 |
| Gupta SK, Singh Z, Purty AJ, Kar M, Vedapriya D, Mahajan P, Cherian J. Diabetes prevalence and its risk factors in rural area of Tamil Nadu. Indian J Community Med. 2010 Jul;35(3):396–9. | 2008 | 1,936 |
| Kumar S, Mukherjee S, Mukhopadhyay P, Pandit K, Raychaudhuri M, Sengupta N, et al. Prevalence of diabetes and impaired fasting glucose in a selected population with special reference to influence of family history and anthropometric measurements-the Kolkata policeman study. J Assoc Physicians India.2008;56:841–4. | 2008 | 2,160 |
| Lau SL, Debarm R, Thomas N, Asha HS, Vasan KS, Alex RG, et al. Healthcare planning in north-east India: a survey on diabetes awareness, risk factors and health attitudes in a rural community. J Assoc Physicians India. 2009 Apr;57:305–9. | 2008 | 144 |
| Vyas U, Khandekar R, Trivedi N, Desai T, Danayak P. Magnitude and determinants of ocular morbidities among persons with diabetes in a project in Ahmedabad, India. (9): 601-7. | 2008 | 40,919 |
| Anjana RM, Pradeepa R, Deepa M, Datta M, Sudha V, Unnikrishnan R, et al. Prevalence of diabetes and prediabetes (impaired fasting glucose and/or impaired glucose tolerance) in urban and rural India: phase I results of the Indian Council of Medical Research-INdia DIABetes (ICMR-INDIAB) study. Diabetologia. 2011 Dec;54(12):3022–7. | 2009 | 14,277 |
| Ravikumar P, Bhansali A, Ravikiran M, Bhansali S, Walia R, Shanmugasundar G, Thakur JS, Kumar Bhadada S, Dutta P. Prevalence and risk factors of diabetes in a community-based study in North India: the Chandigarh Urban Diabetes Study (CUDS). Diabetes Metab. 2011 Jun;37(3):216–21. | 2009 | 2,227 |
| Walia, R et al. High prevalence of cardiovascular risk factors in Asian Indians: A community survey - Chandigarh Urban Diabetes Study (CUDS). Indian Journal of Medical Research. Feb2014, Vol. 139 Issue 2, p252-259. 8p | 2009 | 2,227 |
| Zaman FA, Pal R, Zaman GS, Swati IA, Kayyum A. Glucose indices, frank and undetected diabetes in relation to hypertension and anthropometry in a South Indian rural population. Indian J Public Health. 2011 Mar;55(1):34–7. | 2009 | 1,370 |
| Joshi SR, Saboo B, Vadivale M, Dani SI, Mithal A, Kaul U, et al. Prevalence of Diagnosed and Undiagnosed Diabetes and Hypertension in India—Results from the Screening India’s Twin Epidemic (SITE) Study. Diabetes Technology & Therapeutics. 2012 Jan 1;14(1):8–15. | 2010 | 15,662 |
| Singh AK, Mani K, Krishnan A, Aggarwal P, Gupta SK. Prevalence, awareness, treatment and control of diabetes among elderly persons in an urban slum of Delhi. Indian J Community Med. 2012 Oct;37(4):236–9. | 2010 | 474 |
| Singh A, Milton PE, Nanaiah A, Samuel P, Thomas N. Awareness and attitude toward diabetes in the rural population of Arunachal Pradesh, Northeast India. Indian J Endocrinol Metab. 2012 Mar;16 Suppl 1:S83–86. | 2011 | 149 |
| Vaz NC, Ferreira AM, Kulkarni MS, Vas FS. Prevalence of diabetes mellitus in a rural population of Goa, India. Natl Med J India. 2011 Jan-Feb;24(1):16-8. | 2011 | 1,266 |
| Rajput R, Rajput M, Singh J, Bairwa M. Prevalence of diabetes mellitus among the adult population in rural blocks of Haryana, India: a community-based study. Metab Syndr Relat Disord. 2012 Dec;10(6):443–6. | 2012 | 2,606 |
|  | Total *N* | 447,481 |
