# Supplementary material for: The Health System and Population Health Implications of Large-Scale Diabetes Screening in India: A Microsimulation Model of Alternative Approaches
Source: PLoS Med. 2015 May 19;12(5):e1001827. doi: 10.1371/journal.pmed.1001827 (PMC4437977; doi:10.1371/journal.pmed.1001827)
Supplement: S2 Table — Estimates are for the year 2015. 95% credible intervals are shown in parentheses. (DOCX) [file pmed.1001827.s004.docx]

S2 Table: Probability of diabetes and undiagnosed diabetes by demographic group. Estimates are for the year 2015. 95% credible intervals are shown in parentheses.

| Demographic group | Prevalence of diabetes (%) | Number of people with diabetes (millions) | Proportion of people with diabetes who are undiagnosed (%) | Number of people with undiagnosed diabetes (millions) | Number of people eligible for screening (millions) |
| --- | --- | --- | --- | --- | --- |
| 25-44 years old | 6.4  (4.2-8.7) | 25.2 (16.5-34.0) | 99.7  (96.6-100.0) | 25.2 (16.5-33.8) | 391.6 (391.0-392.2) |
| 45-65 years old | 23.2  (15.2-31.2) | 45.2 (29.6-60.7) | 57.7  (55.9-59.5) | 26 (17.1-35.0) | 175.3 (168.6-182.0) |
| Male | 12.3  (9.4-15.2) | 37.2 (28.3-46.0) | 63.5  (58.4-68.5) | 23.6 (18.7-28.5) | 289.3 (285.0-293.7) |
| Female | 11.7  (7.5-16) | 33.2 (21.2-45.2) | 83.1  (81.3-84.9) | 27.6 (17.2-38) | 277.5 (275.9-279.1) |
| High income | 13.3  (9.4-17.2) | 23.2 (16.3-30.1) | 59.7  (57.7-61.8) | 13.9 (9.8-18) | 165.3 (162.5-168.1) |
| Middle income | 10.6  (7.5-13.8) | 20.4 (14.3-26.4) | 69.7  (67.3-72.1) | 14.2 (10.0-18.4) | 185.5 (183.6-187.3) |
| Low income | 12.2  (8.6-15.8) | 26.8 (18.9-34.8) | 86.3  (83.3-89.3) | 23.1 (16.3-30) | 216.1 (214.9-217.3) |
| Urban | 18.6  (13.1-24.1) | 23.2 (16.4-30.1) | 72  (69.5-74.5) | 16.7 (11.8-21.7) | 118.3 (116.3-120.3) |
| Migrant | 19.3  (13.6-25) | 12.9 (9.1-16.8) | 54.9  (53-56.8) | 7.1 (5.0-9.2) | 61.3 (59.6-63.1) |
| Rural | 8.7  (6.1-11.2) | 34.2 (24.1-44.3) | 80.0  (77.2-82.8) | 27.4 (19.3-35.5) | 387.3 (385.1-389.4) |
| Overall | 12.0  (8.4-15.6) | 70.4 (49.6-91.2) | 73.3  (69.9-76.7) | 51.2 (35.9-66.5) | 566.9 (561.0-572.8) |
