# Supplementary material for: The Health System and Population Health Implications of Large-Scale Diabetes Screening in India: A Microsimulation Model of Alternative Approaches
Source: PLoS Med. 2015 May 19;12(5):e1001827. doi: 10.1371/journal.pmed.1001827 (PMC4437977; doi:10.1371/journal.pmed.1001827)
Supplement: S3 Table — (DOCX) [file pmed.1001827.s005.docx]

S3 Table. Unit component expenditures included in the cost analysis. All costs are expressed in 2014 US Dollars and tabulated over a standard 10-year planning horizon at a 3% annual discount rate. Integrated costs for each screening strategy are tabulated in Table 3.

| *Item* | *Unit cost including overhead (95% CI, 2014 $US)* |
| --- | --- |
| Personnel (cost per annum) | |
| National program manager | $24856.88 ($17399.82-$32313.94) |
| State administration officer | $4522.29 ($3165.6-$5878.98) |
| State clerical officer | $6024.13 ($4216.89-$7831.37) |
| State data entry clerk | $1689.25 ($1182.48-$2196.03) |
| National medical officer | $6661.28 ($4662.9-$8659.66) |
| State Nursing Director/Manager | $3231.33 ($2261.93-$4200.73) |
| State Registered Nurse | $2389.49 ($1672.64-$3106.34) |
| District Health Worker | $1263.18 ($884.23-$1642.13) |
| Operations (cost per annum) | |
| National office and training space | $51547.5 ($36083.25-$67011.75) |
| Provincial office and training space | $6185.7 ($4329.99-$8041.41) |
| Transport for health workers | $4462.5 ($3123.75-$5801.25) |
| Utilities per office | $819.11 ($573.38-$1064.84) |
| Materials (cost per test) | |
| Questionnaires (printing, supplies cost per questionnaire) | $0.05 ($0.04-$0.07) |
| POC glucose test (including per-use meter cost equivalent, and glucometer strip) | $0.43 ($0.3-$0.56) |
| Confirmatory blood glucose testing (including material and personnel costs for blood draw and per-test cost of equipment and reagents) | $2.44 ($1.71-$3.17) |
