# Supplementary material for: The Health System and Population Health Implications of Large-Scale Diabetes Screening in India: A Microsimulation Model of Alternative Approaches
Source: PLoS Med. 2015 May 19;12(5):e1001827. doi: 10.1371/journal.pmed.1001827 (PMC4437977; doi:10.1371/journal.pmed.1001827)
Supplement: S4 Table — (DOCX) [file pmed.1001827.s006.docx]

S4 Table: Comparison of model estimates to three independent estimates for all available demographic groups and years of data [17–19]. 95% credible intervals are shown in parentheses.

| *Data source* | *Year* | *Population* | *Statistic* | *Independent estimate (95% CI)* | *Model estimate (95% CI)* |
| --- | --- | --- | --- | --- | --- |
| Global Burden of Metabolic Risk Factors of Chronic Disease Working Group [17] | 1980 | Men | Total diabetes prevalence | 0.080 (0.031-0.150) | 0.080 (0.061-0.098) |
|  | 1990 | Men | Total diabetes prevalence | 0.083 (0.059-0.110) | 0.084 (0.072-0.096) |
|  | 2000 | Men | Total diabetes prevalence | 0.097 (0.078-0.117) | 0.095 (0.075-0.115) |
|  | 2008 | Men | Total diabetes prevalence | 0.111 (0.081-0.144) | 0.108 (0.070-0.145) |
|  | 1980 | Women | Total diabetes prevalence | 0.075 (0.028-0.143) | 0.076 (0.049-0.102) |
|  | 1990 | Women | Total diabetes prevalence | 0.082 (0.059-0.107) | 0.084 (0.066-0.101) |
|  | 2000 | Women | Total diabetes prevalence | 0.097 (0.079-0.118) | 0.095 (0.065-0.124) |
|  | 2008 | Women | Total diabetes prevalence | 0.108 (0.079-0.141) | 0.106 (0.051-0.160) |
| WHO SAGE [18] | 2003 | 25-44 yr olds | Diagnosed diabetes prevalence | 0.012 (0.008-0.015) | 0.014 (0.010-0.017) |
|  | 2003 | 45-64 yr olds | Diagnosed diabetes prevalence | 0.034 (0.027-0.041) | 0.050 (0.037-0.063) |
|  | 2003 | Men | Diagnosed diabetes prevalence | 0.024 (0.019-0.030) | 0.026 (0.019-0.033) |
|  | 2003 | Women | Diagnosed diabetes prevalence | 0.020 (0.016-0.025) | 0.025 (0.018-0.032) |
|  | 2003 | Urban | Diagnosed diabetes prevalence | 0.054 (0.044-0.065) | 0.040 (0.030-0.050) |
|  | 2003 | Rural | Diagnosed diabetes prevalence | 0.019 (0.015-0.023) | 0.018 (0.014-0.023) |
|  | 2009 | 25-44 yr olds | Diagnosed diabetes prevalence | 0.019 (0.014-0.025) | 0.014 (0.007-0.022) |
|  | 2009 | 45-64 yr olds | Diagnosed diabetes prevalence | 0.047 (0.041-0.054) | 0.053 (0.026-0.081) |
|  | 2009 | Men | Diagnosed diabetes prevalence | 0.040 (0.032-0.048) | 0.028 (0.013-0.042) |
|  | 2009 | Women | Diagnosed diabetes prevalence | 0.018 (0.014-0.022) | 0.027 (0.013-0.040) |
|  | 2009 | Urban | Diagnosed diabetes prevalence | 0.036 (0.027-0.045) | 0.043 (0.021-0.065) |
|  | 2009 | Rural | Diagnosed diabetes prevalence | 0.028 (0.023-0.032) | 0.020 (0.009-0.030) |
| International Diabetes Federation [19] | 2013 | Men | Total population with diabetes | 34,516,820 (no confidence intervals published) | 35,853,689 (19,795,188-51,901,753) |
|  | 2013 | Women | Total population with diabetes | 30,559,540 (no confidence intervals published) | 32,270,645 (10,552,952-53,983,855) |
