# Supplementary material for: The Health System and Population Health Implications of Large-Scale Diabetes Screening in India: A Microsimulation Model of Alternative Approaches
Source: PLoS Med. 2015 May 19;12(5):e1001827. doi: 10.1371/journal.pmed.1001827 (PMC4437977; doi:10.1371/journal.pmed.1001827)
Supplement: S5 Table — (DOCX) [file pmed.1001827.s007.docx]

S5 Table: Sensitivity analysis of large-scale diabetes screening, targeted to different demographic groups. The table displays the number of total positive screens by instrument and by demographic characteristics, providing an estimate of the millions of people sent to the health system for confirmatory testing, and the estimated number of people (in millions) subsequently diagnosed as having diabetes by laboratory assessment of fasting venous glucose. 95% credible intervals are shown in parentheses.

| Instrument: | *Chaturvedi risk score* | *Mohan risk score (“Indian Diabetes Risk Score”)* | *Ramachandran risk score* | *Random POC Glucose (>6.1mmol/L)* | *Chaturvedi risk score then Random POC Glucose (>6.1mmol/L)* | *Mohan risk score (“Indian Diabetes Risk Score”) then Random POC Glucose (>6.1mmol/L)* | *Ramachandran risk score then Random POC Glucose (>6.1mmol/L)* |
| --- | --- | --- | --- | --- | --- | --- | --- |
| Instrument # | 1 | 2 | 3 | 4 | 5 | 6 | 7 |
| Number of people sent for confirmatory testing (millions) | | | | | | | |
| 25-44 yrs old | 179.4 (177.3-181.5) | 149.8 (148-151.6) | 216.6 (214.9-218.3) | 111.8 (109.8-113.9) | 109.4 (107.5-111.3) | 62.9 (60.9-64.8) | 104.7 (103.1-106.3) |
| 45-65 yrs old | 74.1 (73.2-75) | 61.7 (61-62.5) | 89.2 (88.4-89.9) | 46.4 (45.5-47.4) | 45.5 (44.6-46.3) | 26.1 (25.3-27) | 43.4 (42.7-44.1) |
| Male | 130.5 (128.9-132) | 108.9 (107.6-110.2) | 157.4 (156.2-158.6) | 81.4 (79.9-82.9) | 79.7 (78.3-81.1) | 45.8 (44.3-47.2) | 76.2 (75-77.4) |
| Female | 123.1 (121.6-124.5) | 102.7 (101.4-103.9) | 148.4 (147.2-149.5) | 76.9 (75.4-78.3) | 75.2 (73.9-76.6) | 43.2 (41.9-44.6) | 71.9 (70.8-73.1) |
| High income | 74.4 (73.5-75.3) | 62.1 (61.3-62.8) | 89.7 (89.0-90.4) | 46.4 (45.5-47.3) | 45.4 (44.6-46.2) | 26.1 (25.3-26.9) | 43.4 (42.8-44.1) |
| Middle income | 84.0 (83.0-8.05) | 70.1 (69.3-71.0) | 101.4 (100.6-102.2) | 52.4 (51.4-53.4) | 51.3 (50.4-52.2) | 29.5 (28.6-30.4) | 49.1 (48.3-49.8) |
| Low income | 95.1 (94-96.3) | 79.3 (78.4-80.3) | 114.7 (113.7-115.6) | 59.5 (58.3-60.6) | 58.2 (57.1-59.2) | 33.4 (32.4-34.5) | 55.6 (54.7-56.5) |
| Urban | 50.4 (49.7-51) | 42.0 (41.4-42.5) | 60.6 (60.1-61.1) | 31.5 (30.9-32.2) | 30.9 (30.3-31.4) | 17.7 (17.2-18.3) | 29.5 (29.0-3.00) |
| Migrant | 26.8 (26.5-27.1) | 22.3 (22.1-22.6) | 32.3 (32.0-32.5) | 16.7 (16.4-17.1) | 16.4 (16.1-16.7) | 9.4 (9.1-9.7) | 15.7 (15.4-15.9) |
| Rural | 176.4 (174.3-178.5) | 147.3 (145.5-149.1) | 212.9 (211.2-214.6) | 110 .0(107.9-112) | 107.6 (105.7-109.5) | 61.8 (59.9-63.7) | 103.0 (101.4-104.6) |
| Overall | 253.5 (250.5-256.5) | 211.6 (208.9-214.2) | 305.8 (303.3-308.2) | 158.3 (155.3-161.3) | 154.9 (152.1-157.6) | 89.0 (86.2-91.8) | 148.1 (145.8-150.4) |
| Number of confirmatory tests that are positive (FBG>7.0 mmol/L) (millions) | | | | | | | |
| 25-44 yrs old | 25.8 (24.2-27.3) | 18 (16.8-19.2) | 23 (21.6-24.3) | 22.2 (20.4-23.9) | 21.9 (20.3-23.5) | 12.3 (10.7-14) | 18.4 (17.1-19.7) |
| 45-65 yrs old | 11.5 (10.9-12.2) | 8.1 (7.5-8.6) | 10.3 (9.7-10.9) | 9.9 (9.1-10.7) | 9.8 (9.1-10.5) | 5.5 (4.8-6.3) | 8.2 (7.7-8.8) |
| Male | 19.0 (17.9-20.2) | 13.3 (12.4-14.2) | 17.0 (16-17.9) | 16.4 (15.1-17.7) | 16.2 (15-17.4) | 9.1 (7.9-10.4) | 13.6 (12.6-14.6) |
| Female | 18.3 (17.2-19.3) | 12.8 (11.9-13.6) | 16.3 (15.3-17.2) | 15.7 (14.5-16.9) | 15.5 (14.4-16.7) | 8.7 (7.6-9.9) | 13 (12.1-14) |
| High income | 10.9 (10.2-11.5) | 7.6 (7.1-8.1) | 9.7 (9.1-10.3) | 9.4 (8.6-10.1) | 9.3 (8.6-9.9) | 5.2 (4.5-5.9) | 7.8 (7.2-8.3) |
| Middle income | 12.2 (11.5-12.9) | 8.5 (7.9-9.1) | 10.9 (10.2-11.5) | 10.5 (9.7-11.3) | 10.4 (9.6-11.1) | 5.8 (5.1-6.6) | 8.7 (8.1-9.3) |
| Low income | 14.2 (13.4-15.1) | 9.9 (9.3-10.6) | 12.7 (11.9-13.4) | 12.2 (11.3-13.2) | 12.1 (11.2-13) | 6.8 (5.9-7.7) | 10.2 (9.4-10.9) |
| Urban | 7.8 (7.3-8.2) | 5.4 (5.1-5.8) | 6.9 (6.5-7.3) | 6.7 (6.2-7.2) | 6.6 (6.1-7.1) | 3.7 (3.2-4.2) | 5.6 (5.2-6) |
| Migrant | 4.0 (3.8-4.3) | 2.8 (2.6-3.0) | 3.6 (3.4-3.8) | 3.5 (3.2-3.7) | 3.4 (3.2-3.7) | 1.9 (1.7-2.2) | 2.9 (2.7-3.1) |
| Rural | 25.5 (24-27) | 17.8 (16.6-19) | 22.7 (21.4-24) | 21.9 (20.2-23.6) | 21.7 (20.1-23.2) | 12.2 (10.5-13.9) | 18.2 (16.9-19.5) |
| Overall | 37.3 (35.1-39.5) | 26.1 (24.3-27.8) | 33.2 (31.3-35.2) | 32.1 (29.6-34.6) | 31.7 (29.4-34) | 17.9 (15.4-20.3) | 26.7 (24.8-28.5) |
