# Supplementary material for: The Health System and Population Health Implications of Large-Scale Diabetes Screening in India: A Microsimulation Model of Alternative Approaches
Source: PLoS Med. 2015 May 19;12(5):e1001827. doi: 10.1371/journal.pmed.1001827 (PMC4437977; doi:10.1371/journal.pmed.1001827)
Supplement: S6 Table — (DOCX) [file pmed.1001827.s008.docx]

S6 Table: Combined impact of survey-based screening instruments with random glucometer testing. All estimates are in units of millions of people. Compare to Table 3. 95% credible intervals are shown in parentheses.

| Instrument: | Outcome: | *Chaturvedi risk score then Random POC Glucose (>6.1mmol/L)* | *Mohan risk score (“Indian Diabetes Risk Score”) then Random POC Glucose (>6.1mmol/L)* | *Ramachandran risk score then Random POC Glucose (>6.1mmol/L)* | *Random POC Glucose (>6.1mmol/L) then Chaturvedi risk score* | *Random POC Glucose (>6.1mmol/L)*  *then*  *Mohan risk score (“Indian Diabetes Risk Score”)* | *Random POC Glucose (>6.1mmol/L) then Ramachandran risk score* | *Random POC Glucose (>6.1mmol/L) OR Chaturvedi risk score* | *Random POC Glucose (>6.1mmol/L)*  *OR*  *Mohan risk score (“Indian Diabetes Risk Score”)* | *Random POC Glucose (>6.1mmol/L) OR Ramachandran risk score* |
| --- | --- | --- | --- | --- | --- | --- | --- | --- | --- | --- |
| Instrument #: |  | 5 | 6 | 7 | 8 | 9 | 10 | 11 | 12 | 13 |
| Population affected: |  |  |  |  |  |  |  |  |  |  |
| People with undiagnosed diabetes (millions) | True positive screens  (percent of people with undiagnosed diabetes screening positive) | 31.7 (31.7-31.7) (62%) | 17.9 (17.9-17.9) (34.9%) | 26.7 (26.6-26.7) (52%) | 31.8 (31.8-31.9) (62.2%) | 24.2 (24.1-24.2) (47.2%) | 30.9 (30.9-30.9) (60.3%) | 37.7 (37.7-37.7) (73.6%) | 40.6 (40.6-40.6) (79.3%) | 38.8 (38.8-38.8) (75.8%) |
|  | False negative screens  (percent of people with undiagnosed diabetes screening negative) | 19.5 (19.5-19.5) (38%) | 33.3 (33.3-33.4) (65.1%) | 24.6 (24.5-24.6) (48%) | 19.4 (19.4-19.4) (37.8%) | 27 (27-27.1) (52.8%) | 20.3 (20.3-20.3) (39.7%) | 13.5 (13.5-13.5) (26.4%) | 10.6 (10.6-10.6) (20.7%) | 12.4 (12.4-12.4) (24.2%) |
| People without diabetes eligible for screening (having previously-unknown diabetes status) (millions) | True negative screens  (percent of people without diabetes screening negative) | 392.5 (392.5-392.5) (76.1%) | 444.6 (444.5-444.6) (86.2%) | 394.2 (394.2-394.2) (76.4%) | 309.8 (309.8-309.8) (60.1%) | 280.7 (280.6-280.7) (54.4%) | 311.7 (311.7-311.7) (60.4%) | 299.4 (299.4-299.5) (58.1%) | 307.6 (307.6-307.6) (59.7%) | 240.9 (240.9-240.9) (46.7%) |
|  | False positive screens  (percent of people without diabetes screening positive) | 123.2 (123.1-123.2) (23.9%) | 71.1 (71.1-71.1) (13.8%) | 121.4 (121.4-121.5) (23.6%) | 205.9 (205.9-205.9) (39.9%) | 235 (235-235) (45.6%) | 204 (204-204) (39.6%) | 216.2 (216.2-216.2) (41.9%) | 208.1 (208-208.1) (40.3%) | 274.8 (274.8-274.8) (53.3%) |
| People referred for confirmatory testing (millions) | Total positive screens  (percent of those screened being referred to confirmatory testing) | 154.9 (154.9-154.9) (27.3%) | 89 (89-89) (15.7%) | 148.1 (148.1-148.1) (26.1%) | 237.7 (237.7-237.7) (41.9%) | 259.2 (259.2-259.2) (45.7%) | 234.8 (234.8-234.8) (41.4%) | 253.9 (253.9-253.9) (44.8%) | 248.6 (248.6-248.6) (43.9%) | 313.6 (313.6-313.6) (55.3%) |
