# Supplementary material for: The Health System and Population Health Implications of Large-Scale Diabetes Screening in India: A Microsimulation Model of Alternative Approaches
Source: PLoS Med. 2015 May 19;12(5):e1001827. doi: 10.1371/journal.pmed.1001827 (PMC4437977; doi:10.1371/journal.pmed.1001827)
Supplement: S7 Table — (DOCX) [file pmed.1001827.s009.docx]

S7 Table: Diabetes complications rates before and after treatment. The table displays the estimated complications rates among undiagnosed persons without the screening program, and under the best-case scenario in which those diagnosed through screening achieve treatment targets (lowering systolic blood pressure down to a target of 140 mmHg, total LDL to <100 mg/dL if not having cardiovascular disease or <70 mg/dL if having cardiovascular disease, and hemoglobin A1c down to 7%) [17]. We additionally simulated the observed impact of behavioral risk factor modification (i.e., lifestyle interventions) to additionally reduce diabetes complications (though not found effective for cardiovascular end-points, such modification appears to potentially reduce the risk of blindness and renal failure) [18,19]. Complications rates are estimated using equations validated among South Asians to estimate 20-year risks for each complication listed below [9,10,15]. The complications rates for those newly-diagnosed versus previously-diagnosed are based on sampling from the corresponding population biomarkers shown in SI Figure S1 using the model to estimate complications rates. The table also displays the minimum number of people necessary to screen and treat (NNST) to prevent one complication among people with previously-undiagnosed diabetes who are newly diagnosed through the large-scale screening process (using random glucometer screening, given its superior performance to the other instruments shown in Table 3), and the number needed to treat (NNT) among those who were previously diagnosed before the large-scale screening over a 20 year time horizon. 95% credible intervals are shown in parentheses. Note that the large difference between the NNST number and a standard NNT number is because of the screening portion of the calculation, which has a large number of false positives and therefore the number needed to *both* screen and treat for previously-undiagnosed diabetes is far greater than just the number needed to treat for previously-diagnosed diabetes to prevent one complication.

| *Diabetes complication* | Column 1 | Column 2 | Column 3 | Column 4 | Column 5 | Column 6 |
| --- | --- | --- | --- | --- | --- | --- |
|  | 20-year risk of complications without screening (%) | 20-year risk of complications if individuals diagnosed because of screening achieve treatment targets for hypertension, hyperlipidemia and hyperglycemia, as well as behavioral modification | Minimum number needed to screen to find a person with previously-undiagnosed diabetes through the highest-specificity screening strategy (random glucometer testing) to prevent one complication event | Minimum NNT among newly-diagnosed persons found through the highest-specificity screening strategy (random glucometer testing) to prevent one complication event (*N* = 158 million screening positive) – *compare to column 6* | Minimum number needed to screen *and then* treat (NNST) among newly-diagnosed persons found through the highest-specificity screening strategy (random glucometer testing) to prevent one complication event over 20 years  (*N* = 158 million screening positive) | Minimum NNT among previously-diagnosed people with diabetes to prevent one complication event over 20 years (*N* =  19 million with previously-diagnosed diabetes) – *compare to column 4* |
| Coronary heart disease | 25.2% (14.0-39.1%) | 23.6 % (13.1-36.6%) | 17.5 (7.75-27.2) – see SI Table S8 | 45.1 (29.1-81.4) | 788.5 (225.4-2,213.0) | 38.9 (25.1-70.2) |
| Stroke | 27.1% (7.6-70.1%) | 23.7% (6.63-61.4%) |  | 120.3 (46.5-431.2) | 2,105.4 (360.7-11,728.3) | 79.1 (30.6-283.3) |
| Blindness | 4.3% (0.8-14.5%) | 3.6% (0.67-11.9%) |  | 258.6 (78.8-1358.2) | 4525.6 (611-36942.6) | 256.1 (76.4-1351.2) |
| Ulcer | 2.1% (0.3-7.6%) | 1.9% (0.3-6.9%) |  | 877.7 (247.2-6095.0) | 15,578.3 (1,915.9-165,782.9) | 1552.1 (430.6-10,610.5) |
| Renal failure | 20.7% (0.1-100.0%) | 17.7% (0.1-100.0%) |  | 201.1 (35.0-48038.1) | 3518.7 (271.5-1306636.0) | 199.16 (34.0-47792.1) |
