# Supplementary material for: The Health System and Population Health Implications of Large-Scale Diabetes Screening in India: A Microsimulation Model of Alternative Approaches
Source: PLoS Med. 2015 May 19;12(5):e1001827. doi: 10.1371/journal.pmed.1001827 (PMC4437977; doi:10.1371/journal.pmed.1001827)
Supplement: S8 Table — (DOCX) [file pmed.1001827.s010.docx]

S8 Table: Performance characteristics of each instrument following re-calibration to the synthetic nationally-representative population (SI Figure S2). See SI Figure S2 for receiver operating characteristic curves. Compare to Table 2, which displays results using cut-points from the published literature, based on ROC analysis from smaller sub-national populations as detailed in Table 1. 95% credible intervals are shown in parentheses.

| *Instrument:* | *Chaturvedi risk score* | | *Mohan risk score*  *(“Indian Diabetes Risk Score”)* | | *Ramachandran risk score* | | *Random POC Glucose)* | |
| --- | --- | --- | --- | --- | --- | --- | --- | --- |
| Instrument # | 1 | | 2 | | 3 | | 4 | |
| New cut-point (see SI Figure S2 for ROC curves) | Score>19 (from 16) | | Score>52 (from 60) | | Score>26 (from 21) | | RPG>6.8mmol/L (from 6.1) | |
| Performance in detecting undiagnosed diabetes | Published estimates from sub-national cohorts | Estimate from model (synthetic national cohort) (%) | Published estimates from sub-national cohorts | Estimate from model (synthetic national cohort) (%) | Published estimates from sub-national cohorts | Estimate from model (synthetic national cohort) (%) | Published estimates from sub-national cohorts | Estimate from model (synthetic national cohort) (%) |
| Sensitivity | 73% (68-77%) in industrial workforce cohort from multiple Indian sites, 2001-2003; 66% (95% CI: 59-73%) in urban Delhi and rural Haryana, 1991–1994; | 58.8 (57.8-59.8) | 73% in urban and rural Chennai, 2001-2002 (no credible intervals reported) | 53.0 (52.1-54.0) | 77% and 72% in two cohorts from six cities (2000); 74% in a cohort from Chennai (1995); 92% in the South Asian cohort from the Health Survey for England (1999) (no credible intervals reported) | 57.5 (56.6-58.4) | 78% in rural Andhra Pradesh (no credible intervals reported) | 62.2 (60.6-63.7) |
| Specificity | 56% (55-57%) in industrial workforce cohort from multiple Indian sites, 2001-2003; 67% (95% CI: 65-68%) in urban Delhi and rural Haryana, 1991–1994 | 79.9 (79.3-80.6) | 60% in urban and rural Chennai, 2001-2002 (no credible intervals reported) | 73.9 (73.4-74.5) | 60% and 59% in two cohorts from six cities (2000); 61% in a cohort from Chennai (1995); 26% in the South Asian cohort from the Health Survey for England (1999) (no credible intervals reported) | 75.9 (75.5-76.4) | 79% in rural Andhra Pradesh (no credible intervals reported) | 91.9 (91.6-92.3) |
| Positive predictive value | 10% (8-12%) in urban Delhi and rural Haryana, 1991–1994;  6% (5-7%) in industrial workforce cohort from multiple Indian sites, 2001-2003 | 33.2 (13.8-52.6) | 17% in urban and rural Chennai, 2001-2002 (no credible intervals reported) | 18.3 (6.20-30.4) | 9% and 8% in two cohorts from six cities (2000); 12% in a cohort from Chennai (1995); 22% in the South Asian cohort from the Health Survey for England (1999) (no credible intervals reported) | 30.2 (9.77-50.7) | 15% in rural Andhra Pradesh (no credible intervals reported) | 44.7 (18.2-71.2) |
| Negative predictive value | 97% (96-98%) in urban Delhi and rural Haryana, 1991–1994;  98% (97-99%) in industrial workforce cohort from multiple Indian sites, 2001-2003 | 91.9 (89.1-94.7) | 95% in urban and rural Chennai, 2001-2002 (no credible intervals reported) | 93.4 (88.9-97.9) | 98% and 98% in two cohorts from six cities (2000); 97% in a cohort from Chennai (1995); 94% in the South Asian cohort from the Health Survey for England (1999) (no credible intervals reported) | 90.8 (86.5-95.0) | 99% in rural Andhra Pradesh (no credible intervals reported) | 95.8 (92.8-98.9) |
| Number needed to screen to detect one previously-undiagnosed person with diabetes | Not reported | 18.8 (8.4-29.1) | Not reported | 20.8 (9.44-32.2) | Not reported | 19.2 (8.69-29.7) | Not reported | 17.5 (7.75-27.2) |
