# Supplementary material for: The Health System and Population Health Implications of Large-Scale Diabetes Screening in India: A Microsimulation Model of Alternative Approaches
Source: PLoS Med. 2015 May 19;12(5):e1001827. doi: 10.1371/journal.pmed.1001827 (PMC4437977; doi:10.1371/journal.pmed.1001827)
Supplement: S1 Text — (DOCX) [file pmed.1001827.s011.docx]

**SI Text S1**

The modeling proceeded in three stages. First, we constructed a synthetic nationally representative Indian population from a series of subnational cohort studies. Second, we subjected each member of the synthetic population to each of three survey-based screening instruments advocated for the detection of people with undiagnosed diabetes in India. Third, we estimated what portion of the true positives, diagnosed upon confirmatory testing with fasting blood glucose, would require treatment to produce significant population-level reductions in the risk of diabetes complications. This SI text provides details of each of these processes in accordance with standard international model reporting guidelines [1].

To construct the synthetic population, a systematic review of the literature was first performed to find prior studies cataloguing the prevalence of total, diagnosed and/or previously-undiagnosed diabetes among any subgroups of the Indian population. We searched the electronic databases Pubmed, IndMED, WHOLIS (WHO Library system) and Google Scholar, using the following combination of terms:

Search #1: "Diabetes"[Title or Abstract word] OR “Diabetes Mellitus”[MeSH Terms];

Search #2: "India"[MeSH Terms] OR "Asia"[MeSH Terms] OR "global"[MeSH Terms] OR “Indian"[Title or Abstract word] OR “South Asian” [Title or Abstract word]

Search #3: #1 AND #2 AND NOT (Letter[ptyp] OR Editorial[ptyp]);

The search was performed in August 2014 and included articles from January 1980 through January 2014. Of note, none of the articles fulfilling the criteria were in non-English languages. All titles and abstracts found by the search strategy were filtered for relevance to the study objective. Studies must have included data on a population in at least one Indian location. The abstracts of potentially-relevant articles were subject to the following inclusion criteria to ensure they met basic minimum methodological standards: new diabetes diagnoses must meet or exceed World Health Organization clinical criteria and laboratory standards for diagnosis [2,3]; sample size must include at least twenty patients per facility or location described, or more than one-hundred persons if community-based household surveys; if questionnaire-based, the study must have at least fifty percent response rate; data description must include data selection criteria, population demographic description, data collection method, and statistical analysis description; for household surveys, study must include census of households and either stratified or random selection from a list of available households; data and tables should add up and be consistent; absolute numbers must be given or denominators must be available for percentage results; and no obvious aspects of the design or analysis, or unusual events in the study, could have occurred that could introduce bias in the prevalence estimates. We excluded data from pregnant women or persons with prenatal diabetes. A data extraction method was designed by two reviewers (SB, RA). RA extracted the data using a pre-established standard data entry format into an Excel spreadsheet, with verification by SB to ensure consistency of coding. Standard data describing each study were also extracted, including study location, study period, study methodology, number of included subjects, primary and secondary outcomes measures and endpoints, and study limitations. Where disclosed, we noted the study funders and agencies. Disagreements between the two reviewers were resolved by consensus among the authors. During the initial search period, 70,545 studies were identified through the database searches, of which 9,845 remained after removal of duplicates, including multiple publications of the same cohort data (including same year prevalence estimates) via multiple publications. These 9,845 were screened and assessed for eligibility through review of titles, and 3,151 potentially-relevant articles were subject to abstract review, from which 58 papers were included in the ultimate quantitative synthesis.

The included studies (detailed in S1 Table) were subject to the mixed-effects meta-regression model specified in the main text to estimate prevalence of total and previously-undiagnosed diabetes and associated risk factors (Table 1) by year, specific to age, sex, income (SLI) and location (urban, migrant and rural). To perform the meta-regression specified in the main text, authors of the included studies (S1 Table) were contacted when clarification on moderator values, definitions, cut-points, or unit conversions was required. Estimates of the model parameters were obtained via a restricted maximum likelihood procedure, including prevalence estimates only once when the same cohort prevalence was reported multiple times (or in multiple publications) for the same year, producing the estimates provided in S3 Table for diabetes prevalence and probability of being undiagnosed, and in S1 Figure for risk factor and disease co-morbidity distributions. Missing data were imputed using the standard nonparametric trim-and-fill method on the baseline random-effects model without moderators before application to the full mixed-effects model [4], checking passage of an Egger regression test for publication bias [5]. Note that we included time in the model after comparing linear, quadratic and secular trends, finding that the linear or quadratic equations were superior to cubic formulations by Akaike’s information criterion (*Δ*AIC>10) [6]. We ensured that estimates passed the generalized/weighted least squares version of Cochran’s QE-test for residual heterogeneity [7], which tests whether the variability in the observed effect sizes or outcomes that is not accounted for by the moderators included in the model is larger than one would expect based on sampling variability.

To construct the simulated population based on these estimates, we simulated the population of India based on UN estimates for the year 2015 [8], dividing the simulated population into cohorts defined by age (years 25 to 44 and 45 to 65, among whom the equations for estimating complication rates have been validated [9,10]), sex, location (urban, rural, or migrant), and income (tertiles of the standard of living index, a standard metric of wealth in India [11]). For each simulated individual, we determined whether they have diabetes in the model by sampling from the meta-regression estimate of total diabetes prevalence corresponding to their age, sex, income and location, using a standard random binomial distribution where the probability of having diabetes is given by a random sample from the normal distribution defined by the mean prevalence and standard deviation around the mean for their age, sex, income and location (SI Table S3). Among those having diabetes, we then repeated a binomial probability sampling process using the sample probability of being undiagnosed specific to that individual’s age, sex, income and location (also in SI Table S3), in order to determine whether the simulated individual with diabetes is previously diagnosed or undiagnosed. For each individual in the model, we then sampled from the kernel smoothing densities describing the prevalence and covariance of each risk factor, conditional on diabetes and diagnosis status, which captures how the risk factor values vary among people with and without diabetes, as well as among diagnosed versus undiagnosed status among those people with diabetes. We accounted for the covariance between risk factor values (SI Figure S1) using standard copula functions [12,13].

Given the risk factor values of each simulated individual, we determined whether the individual would cross the threshold to be detected as “high risk” among each of survey-based screening instruments. We sampled from the blood glucose testing distributions from the IMS Study to assign simulated individuals a random blood glucose value, again specific to their age, sex, income and location as well as conditional on their diabetes and diagnosis status and using a copula to account for covariance between random glucose and other risk features (SI Figure S1). We added to this value the estimated error of point-of-care glucometer testing, by sampling from the distribution *N(0,0.81)* mmol/L, which reflects the error in a prior Indian field trial in which random glucometer testing was compared to gold-standard laboratory venous glucose tests [14]. We then determined which persons would exceed the threshold of >6.1 mmol/L, to assess the performance of the random glucometer screening as compared to the survey-based screening instruments. We tallied the number of people with true undiagnosed diabetes versus those without diabetes who would be classified as “high risk” by each of the screening instruments or random glucometer screening to tabulate true and false positives, and similarly tabulated those who failed to be listed as “high risk” to tabulate true and false negatives and subsequent sensitivity and specificity.

The next task in our model was to estimate the rate of incidence among the simulated population associated with each of five major diabetes complications. We used the UKPDS risk equations, which are published in full detail previously [9,10,15]. The parameters for each equation that were used in our model were those specific to South Asians. To check for accurate reproduction of the equations, we ensured that our calculated risk estimates matched the examples provided in prior UKPDS publications [9,10,15]. The equations are validated among the South Asian population in the UKPDS study, where the outcomes are defined specifically per the original UKPDS trial. In particular, coronary heart disease was defined as fatal or first non-fatal myocardial infarction [9]. Stroke was defined as first neurological deficit with symptoms or signs lasting 1 month or more, including ischemic, embolic, and hemorrhagic strokes [10]. Renal failure was defined as plasma creatinine greater than 250 micromol/L (2.8 mg/dL) or hemodialysis unrelated to any acute intercurrent illness [15]. In order to estimate the value of each risk equation, we sampled from the IMS study to provide individuals with biomarker values specific to their age, sex, income and location conditional on diabetes and diagnosis status, again using copulas to account for covariance among these factors as observed in the IMS (S1 Figure). We calculated the risk of each simulated individual before any intervention (i.e., with no change in their biomarker values), and after meeting the ADA targets of systolic blood pressure <140 mmHg, LDL < 100 mg/dL (or <70 mg/dL if having a prior history of coronary heart disease or stroke), and hemoglobin A1c<7%. In the case of low-density lipoprotein (LDL), the UKPDS risk equations use total/HDL cholesterol in some equations; in this case, we calculated the change in total/HDL anticipated given the change in absolute LDL, and entered this value into the equations. We similarly calculated the estimated change in probability of microalbuminuria given reduction in systolic pressure for renal failure, which is an estimated 44% reduction for achievement of the systolic pressure target (95% CI: 39-50%) [16]. These inclusions were meant to give the most optimistic effect from treatment, providing an estimate of the minimum number needed to treat to prevent one diabetes complication.

Lastly, we computed the receiver operating characteristic (ROC) curves for each instrument by varying the cut-points considered positive and plotting 1 minus the specificity of each instrument against its sensitivity (S2 Figure). We found the optimal cut-point for each instrument and compared it to the cut-point chosen through previous studies among sub-national populations. We then compared the change in sensitivity, specificity, positive predictive value, negative predictive value, and number needed to screen among the recalibrated instruments versus the instruments using the older cut-points (S8 Table).

In order to produce a comprehensive multivariate uncertainty analysis, we ran the model repeatedly while sampling from the probability distributions of all input parameters. We found that the 95% credible intervals around model outcomes were stable after 10,000 iterations of repeated sampling.

**S1 Text References**

1. Caro JJ, Briggs AH, Siebert U, Kuntz KM. Modeling Good Research Practices—Overview: A Report of the ISPOR-SMDM Modeling Good Research Practices Task Force–1. Med Decis Making. 2012;32: 667–677.

2. World Health Organization. Use of Glycated Haemoglobin (HbA1c) in the Diagnosis of Diabetes Mellitus. Geneva: WHO; 2011.

3. World Health Organization. Definition and diagnosis of diabetes mellitus and intermediate hyperglycemia : report of a WHO/IDF consultation. Geneva: WHO; 2006.

4. Duval S, Tweedie R. A nonparametric “trim and fill” method of accounting for publication bias in meta-analysis. J Am Stat Assoc. 2000;95: 89–98.

5. Egger M, Smith GD, Schneider M, Minder C. Bias in meta-analysis detected by a simple, graphical test. Bmj. 1997;315: 629–634.

6. Akaike H. Information theory and an extension of the maximum likelihood principle. Selected Papers of Hirotugu Akaike. Springer; 1998. pp. 199–213. Available: http://link.springer.com/chapter/10.1007/978-1-4612-1694-0_15

7. Viechtbauer W. Conducting meta-analyses in R with the metafor package. J Stat Softw. 2010;36: 1–48.

8. United Nations. World Population Prospects: The 2012 Revision. Geneva: UN; 2013.

9. Stevens RJ, Kothari V, Adler AI, Stratton IM, Holman RR, Group UKPDS (UKPDS), et al. The UKPDS risk engine: a model for the risk of coronary heart disease in Type II diabetes (UKPDS 56). Clin Sci. 2001;101: 671–679.

10. Kothari V, Stevens RJ, Adler AI, Stratton IM, Manley SE, Neil HA, et al. UKPDS 60 risk of stroke in type 2 diabetes estimated by the UK Prospective Diabetes Study risk engine. Stroke. 2002;33: 1776–1781.

11. International Institute for Population Sciences. National Family Health Survey, India 2005-06. Bombay: IIPS; 2008.

12. Mai J-F, Scherer M. Simulating Copulas: Stochastic Models, Sampling Algorithms, and Applications. Imperial College Press; 2012.

13. Hofert M, Mächler M. Nested Archimedean copulas meet R: The nacopula package. J Stat Softw. 2011;39: 1–20.

14. Ritchie GE, Kengne AP, Joshi R, Chow C, Neal B, Patel A, et al. Comparison of near-patient capillary glucose measurement and a risk assessment questionnaire in screening for type 2 diabetes in a high-risk population in rural India. Diabetes Care. 2011;34: 44–49.

15. Hayes AJ, Leal J, Gray AM, Holman RR, Clarke PM. UKPDS Outcomes Model 2: a new version of a model to simulate lifetime health outcomes of patients with type 2 diabetes mellitus using data from the 30 year United Kingdom Prospective Diabetes Study: UKPDS 82. Diabetologia. 2013; 1–9.

16. Viberti G, Wheeldon NM. Microalbuminuria Reduction With Valsartan in Patients With Type 2 Diabetes Mellitus A Blood Pressure–Independent Effect. Circulation. 2002;106: 672–678. doi:10.1161/01.CIR.0000024416.33113.0A

17. Danaei G, Finucane MM, Lu Y, Singh GM, Cowan MJ, Paciorek CJ, et al. National, regional, and global trends in fasting plasma glucose and diabetes prevalence since 1980: systematic analysis of health examination surveys and epidemiological studies with 370 country-years and 2.7 million participants. Lancet. 2011;378: 31–40. doi:10.1016/S0140-6736(11)60679-X

18. Vellakkal S, Subramania SV, Millett C, Basu S, Stuckler D, Ebrahim S. Socioeconomic inequalities in non-communicable diseases prevalence in India: Disparities between self-reported diagnoses and standardized measures. PLoS ONE. in press.

19. International Diabetes Federation. Diabetes Atlas. 6th edition. Brussels: IDF; 2013.
